# Glycerol bioconversion to biofuel and value-added products by yeasts

**DOI:** 10.1093/femsyr/foaf038

**Published:** 2025-07-22

**Authors:** Kostyantyn Dmytruk, Marta Semkiv, Andriy Sibirny

**Affiliations:** Department of Molecular Genetics and Biotechnology, Institute of Cell Biology, NAS of Ukraine, Drahomanov Street, 14/16, Lviv 79005, Ukraine; Leibniz Institute of Plant Genetics and Crop Plant Research (IPK), Corrensstr. 3, OT Gatersleben, D-06466 Seeland, Germany; Department of Molecular Genetics and Biotechnology, Institute of Cell Biology, NAS of Ukraine, Drahomanov Street, 14/16, Lviv 79005, Ukraine; Faculty of Biotechnology, Medical College, University of Rzeszów, Cwiklinskiej 2D, Rzeszów 35-601, Poland

**Keywords:** glycerol, yeast, bioconversion, biofuel, organic acids, polyols

## Abstract

Glycerol, a by-product of biodiesel production, is a versatile polyol used in various industries. Yeasts play a crucial role in converting glycerol into biofuels and value-added products, offering sustainable alternatives to chemical synthesis. This review explores glycerol metabolism in yeasts, focusing on its bioconversion into ethanol, isopropanol, lipids, organic acids, and polyols. *Saccharomyces cerevisiae* and *Yarrowia lipolytica* are prominent species for these processes, with metabolic engineering enhancing their efficiency. Ethanol production from crude glycerol, a by-product of the biodiesel industry, is cost-effective compared to traditional feedstocks, while lipid production by oleaginous yeasts supports biodiesel synthesis. Organic acids like succinic, citric, and lactic acids, along with polyols such as erythritol and mannitol, are produced through optimized pathways, achieving high yields. Crude glycerol, despite impurities, is a viable low-cost substrate, with yeast strains adapted to tolerate its contaminants. Challenges include improving strain tolerance and scaling up processes. Future research aims to refine metabolic engineering and fermentation strategies to maximize glycerol’s potential as a renewable feedstock for industrial biotechnology.

## Teaser

Yeasts can transform waste glycerol from biodiesel production into ecofriendly fuels and valuable products, offering a sustainable and cost-effective alternative to traditional chemical manufacturing methods.

## Introduction

Glycerol is a simple polyol that serves as a major component of storage lipids (triacylglycerols) and phospholipids in cell membranes (Ali and Szabó 2023). In yeast cells, free glycerol accumulates primarily as an osmolyte (de Nadal and Posas 2022). Due to its versatile properties, glycerol is widely utilized across various industries, including food, pharmaceuticals, cosmetics, toothpaste, paints, drugs, paper, textiles, leather, and explosives (Chilakamarry et al. 2021). It can be produced chemically from oils (triacylglycerides) or propylene, as well as through microbial synthesis, with yeasts emerging as promising producers (Semkiv et al. 2020). In recent years, the biodiesel industry has generated a substantial amount of glycerol as a by-product—commonly referred to as crude glycerol. Global production of crude glycerol, glycerine waters, and lyes reached ~7.6 million tons in 2024 (IndexBox 2024). However, crude glycerol is typically contaminated with methanol, salts, and other impurities, posing challenges for its direct application. This review explores glycerol metabolism in yeasts and the bioconversion of both purified and crude glycerol into bulk and value-added chemicals.

## Yeast metabolism of glycerol

**Figure 1.**
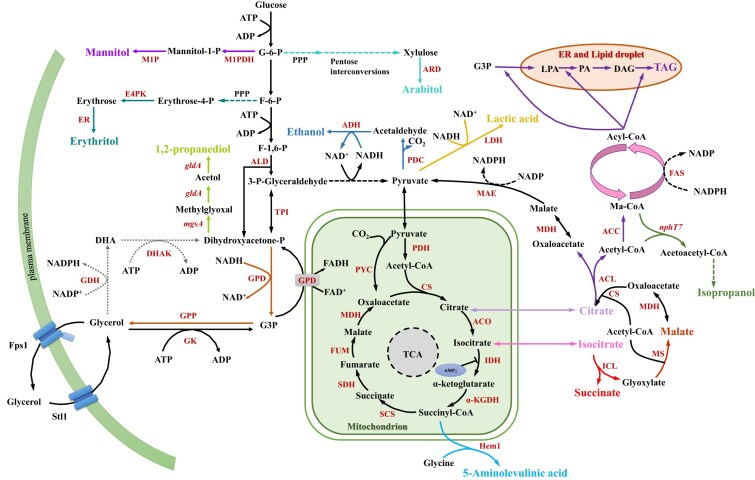
Glycerol metabolism and bioconversion. Routes to different end-products are marked with different colors. Abbreviations, metabolites: DAG—diacylglycerol, DHA—dihydroxyacetone, F-6-P—fructose-6-phosphate, F-1,6-P—fructose-1,6-biphosphate, G3P—glycerol-3-phosphate, G-6-P—glucose-6-phosphate, LPA—lysophosphatidic acid, PA—phosphatidic acid, and TAG—triacylglycerol; pathways: PPP—pentose phosphate pathway and TCA—tricarboxylic acid cycle; enzymes: ACC—acetyl-CoA carboxylase, ACL—ATP citrate lyase, ACO—aconitase, ADH—alcohol dehydrogenase, ALD—aldolase, ARD—arabitol dehydrogenase, CS—citrate synthase, DHAK—dihydroxyacetone kinase, E4PK—erythrose-4-phosphate kinase, ER—erythrose reductase, FAS—fatty acid synthetase, FUM—fumarase, GDH—glycerol dehydrogenase, GK—glycerol kinase, gldA—glycerol dehydrogenase from *E. coli*, GPD—glycerol-3-phosphate dehydrogenase, GPP—glycerol-3-phosphate phosphatase, Hem1–5-aminolevulinic acid synthase from *S. cerevisiae*, ICL—isocitrate lyase, IDH—isocitrate dehydrogenase, KGDH—α-ketoglutarate dehydrogenase, LDH—lactate dehydrogenase, MAE—malic enzyme, MDH—malate dehydrogenase, mgsA—methylglyoxal synthase from *E. coli*, M1P—mannitol-1-phosphatase, M1PDH—mannitol-1-phosphate dehydrogenase, MS—malate synthase, nphT7–acetoacetyl-CoA synthase from *Streptomyces* sp., PDC—pyruvate decarboxylase, PDH—pyruvate dehydrogenase, PYC—pyruvate carboxylase, α-SCS—succinyl-CoA synthetase, SDH—succinic dehydrogenase, TPI—triose phosphate isomerase; Stl1, Fps1–membrane transporters.

Yeasts are capable of both producing and utilizing glycerol. Glycerol metabolism has been most thoroughly characterized in *Saccharomyces cerevisiae* (Fig. 1), while information on other yeasts remains limited. In *S. cerevisiae*, glycerol is synthesized from the glycolytic intermediate dihydroxyacetone phosphate (DHAP) through two cytosolic enzymes: glycerol-3-phosphate dehydrogenase (GPD) and glycerol-3-phosphate phosphatase (GPP). DHAP and glyceraldehyde-3-phosphate (GAP) originate from the cleavage of fructose-1,6-bisphosphate by aldolase and can interconvert with each other via triose phosphate isomerase (TPI).

The NAD⁺-dependent GPD, which catalyzes the reduction of DHAP to glycerol-3-phosphate (G3P), is the rate-limiting step in glycerol synthesis (Remize et al. 2001). It is encoded by two isogenes, *GPD1* and *GPD2*, whose protein products share 69% identity (Eriksson et al. 1995). Despite their similarity, they serve different functions: *GPD1* is crucial under osmotic stress and activated by the high osmolarity glycerol (HOG) pathway (Albertyn et al. 1994), while *GPD2* plays a key role in maintaining redox balance during anaerobic growth (Ansell et al. 1997). A *gpd1Δ gpd2Δ* double mutant cannot produce glycerol and fails to grow in high-osmolarity environments or grow anaerobically unless supplemented with an NADH-oxidizing compound (Ansell et al. 1997, Bjorkqvist et al. 1997).

GPD genes have also been identified in other yeasts, including *Starmerella magnoliae* (Lee et al. 2008), *Pichia kudriavzevii*, and *Debaryomyces hansenii*. Some species, such as *Wickerhamiella versatilis*, also exhibit NADP⁺-dependent GPD activity (Watanabe et al. 2008).

The second step in glycerol synthesis involves the irreversible hydrolysis of the phosphate group from G3P, catalyzed by GPP (Norbeck and Blomberg 1997). Two genes, *GPP1* and *GPP2*, encode highly similar isoenzymes with 95% sequence identity. Their expression is differentially regulated, mirroring the regulation of GPD genes. *GPP1* expression is induced under anaerobic conditions, and *gpp1Δ* mutants show impaired anaerobic growth (Pahlman et al. 2001). In contrast, *GPP2* expression is strongly activated by the Hog1 MAP kinase in response to osmotic stress (Norbeck and Blomberg 1997, Pahlman et al. 2001). Single *gpp1Δ* or *gpp2Δ* mutants do not show growth defects under osmotic stress, suggesting functional redundancy. However, the *gpp1Δ gpp2Δ* double mutant exhibits growth inhibition under anaerobic conditions, heightened sensitivity to osmotic stress, accumulation of G3P, and reduced glycerol production (Pahlman et al. 2001).

Glycerol is abundant in nature, and many microorganisms can use it as a carbon and energy source. Its utilization occurs via two main pathways: one through G3P and another via dihydroxyacetone (DHA). In the G3P pathway, glycerol is phosphorylated by glycerol kinase (GK) and oxidized by FAD-dependent GPD, which is located on the outer surface of the inner mitochondrial membrane. This GPD transfers electrons directly to the respiratory chain. In *S. cerevisiae*, this is the primary glycerol utilization route, with GK and GPD encoded by *GUT1* and *GUT2*, respectively (Fig. 1) (Sprague and Cronan 1977, Pavlik et al. 1993, Ronnow and Kielland-Brandt 1993). Mutants lacking either gene cannot grow on glycerol as the sole carbon source.

The mitochondrial Gut2, along with cytosolic Gpd1 and Gpd2, also contributes to the G3P shuttle, which helps regenerate NAD⁺ from NADH during anaerobic growth (Larsson et al. 1998). The G3P pathway has also been identified in other yeasts, such as *D. hansenii, Zygosaccharomyces rouxii*, and *P. kudriavzevii* (Adler et al. 1985, van Zyl et al. 1991, Wang et al. 2000).

The alternative DHA pathway begins with the oxidation of glycerol to DHA by glycerol dehydrogenase (GDH), encoded by genes *GCY1/YPR1*, followed by phosphorylation by dihydroxyacetone kinase (DHAK), encoded by *DAK1* and *DAK2*. In *S. cerevisiae*, this route plays a minor role, as it cannot compensate for the loss of the G3P pathway in *gut1Δ* or *gut2Δ* mutants. However, a synthetic, NAD⁺-dependent DHA pathway has been engineered to substitute the endogenous FAD-dependent G3P route. This modification facilitated *S. cerevisiae* growth on glycerol and enabled the potential formation of fermentation products. (Klein et al. 2016a). In other yeasts, the DHA pathway may be more prominent.

Tani and Yamada (1987) categorized yeast species based on the presence of GK or NAD⁺-dependent GDH activity into three groups: (1) those using only the G3P pathway (e.g. *Candida boidinii*), (2) those using only the DHA pathway (e.g. *Zygoascus ofunaensis*), and (3) those capable of both (e.g. *Pichia membranifaciens*). However, enzyme activity alone does not confirm pathway functionality. Stronger evidence includes the loss of glycerol utilization upon deletion of a key gene, as shown in *S. cerevisiae gut1Δ* or *gut2Δ* strains. Similarly, in *Schizosaccharomyces pombe*, deletion of *gld1* reduced NAD⁺-dependent GDH activity and abolished growth on glycerol (Matsuzawa et al. 2010).

Although much of the data on glycerol utilization comes from *S. cerevisiae*, most *S. cerevisiae* strains display poor growth on media where glycerol is the sole carbon source. However, their growth can be enhanced by supplementing the medium with amino acids, nucleobases, or complex nutrients (Merico et al. 2011, Swinnen et al. 2013). In contrast, several other yeast species exhibit superior glycerol utilization. For instance, in a study testing 42 yeast species, *Cyberlindnera jadinii* and *Wickerhamomyces anomalus* achieved growth rates approximately three times higher than *S. cerevisiae* (Lages et al. 1999).

Yeasts such as *Yarrowia lipolytica, Komagataella phaffii*, and *Pachysolen tannophilus* are now employed in industrial processes using glycerol-based media, showing growth rates 1.3–3.3 times higher than the best-performing *S. cerevisiae* strain (Klein et al. 2016b). Glycerol metabolism in *Y. lipolytica* can be further enhanced by overexpressing *GUT1* (Carly et al. 2017, Ong et al. 2020), or by coexpressing *S. cerevisiae* sugar alcohol phosphatase (PYP) along with its native GK and transketolase (TKL) (Jagtap et al. 2021).

To serve as a carbon source, glycerol must first be transported into the cell. Conversely, excess glycerol—such as that produced during anaerobic NAD⁺ regeneration—must be exported. In *S. cerevisiae*, glycerol flux across the plasma membrane is well characterized. Ferreira et al. (2005) identified the glycerol/H⁺ symporter Stl1 as essential for active glycerol uptake; deletion of *STL1* abolishes this transport and prevents growth on glycerol as the sole carbon source. *STL1* expression is transiently induced under hyperosmotic conditions (Posas et al. 2000, Ferreira et al. 2005) but decreases as intracellular glycerol synthesis is activated. This downregulation is absent in *gpd1Δ gpd2Δ* mutant, which cannot produce glycerol (Ferreira et al. 2005). Similar H⁺- or Na⁺-coupled glycerol symporters have been described in halotolerant yeasts such as *D. hansenii, Millerozyma farinosa* (formerly *Pichia sorbitophila*), and *Z. rouxii* (Lucas et al. 1990, van Zyl et al. 1991, Lages and Lucas 1995).

Glycerol export in *S. cerevisiae* is mediated by the aquaglyceroporin Fps1 (Luyten et al. 1995), which closes at high osmolarity to retain intracellular glycerol (Tamas et al. 1999). Fps1 closure is among the earliest responses to osmotic shock (Petelenz-Kurdziel et al. 2013). Upon a shift to low osmolarity, Fps1 reopens and releases accumulated glycerol within minutes. *fps1Δ* mutants are highly sensitive to such shifts, highlighting the channel’s role in hypoosmotic stress protection (Luyten et al. 1995, Tamas et al. 1999). Under anaerobic conditions, *FPS1* expression is upregulated to export excess glycerol formed during NADH reoxidation (ter Linde et al. 1999).

Fps1 has a core domain flanked by long N- and C-terminal extensions (∼230–250 amino acids) required for channel closure. Deletion of either domain results in constitutive channel activity, leading to continuous glycerol efflux and compensatory overproduction (Ahmadpour et al. 2014). Similar effects are caused by mutations preventing phosphorylation of Thr231 in the N-terminal extension. Lee et al. (2013) showed that Fps1 closure is triggered by phosphorylation of Rgc2; in its unphosphorylated form, Rgc2 binds to the Fps1 C-terminus, keeping the channel open. Additionally, the MAPK Slt2, activated by hypoosmotic shock and cell wall stress, may regulate rapid Fps1 opening (Levin 2011, Baltanas et al. 2013). Notably, this complex regulation appears limited to yeasts closely related to *S. cerevisiae* (Pettersson et al. 2005), likely as an adaptation to sudden osmotic changes. In contrast, osmotolerant yeasts such as *D. hansenii* and *M. farinosa* lack Fps1, possibly to prevent glycerol leakage in high-osmolarity environments (Prista et al. 2005, Sabir et al. 2016).

Homologs of *STL1* and *FPS1* have been identified in efficient glycerol-utilizing yeasts *Y. lipolytica, P. tannophilus*, and *K. phaffii* (Dujon et al. 2004, Mattanovich et al. 2009, Liu et al. 2013). Their roles in glycerol metabolism, however, remain unclear. Interestingly, in contrast to *S. cerevisiae*, some Fps1 homologs in other yeasts may function in glycerol uptake. For example, expression of *FPS2* from *P. tannophilus* in *S. cerevisiae* restored growth on glycerol in a *stl1Δ* mutant, whereas overexpressing native *FPS1* did not (Liu et al. 2013). Structurally, PtFps2 lacks the long terminal extensions of ScFps1, containing only the six predicted transmembrane domains. Overexpression of *PtFPS2* and similar *FPS1* homologs from nonconventional yeasts like *C. jadinii, K. phaffii*, and *Y. lipolytica* significantly enhanced growth on glycerol in *S. cerevisiae*, even in the absence of *STL1* (Klein et al. 2016a).

## Glycerol bioconversion to biofuels

Global biodiesel production has been rapidly increasing as a sustainable alternative to petroleum (Farouk et al. 2024). A significant by-product of this process is glycerol, which accounts for roughly 10% of biodiesel output by weight. It is generated via methanol-based transesterification reactions catalyzed by NaOH or KOH (Garlapati et al. 2016). The economic viability of biodiesel production strongly depends on the effective utilization of this surplus glycerol, driving global interest in its value-added applications (Willke and Vorlop 2008).

Glycerol serves as a versatile feedstock for the chemical synthesis of a wide range of products, including glyceric acid, DHA, ketomalonic acid, poly(ketomalonate), 1,2,3-tri-tert-butyl glycerol, propylene glycol, acrolein, epichlorohydrin, and others (Pagliaro et al. 2007). However, traditional chemical methods often require toxic reagents and generate environmentally harmful by-products. In contrast, biological conversion of glycerol—particularly crude glycerol from biodiesel production—into biofuels and other high-value compounds using yeasts offers a greener and more sustainable alternative.

Glycerol is more reduced than sugars on a per-carbon basis, meaning its metabolism yields more NADH than glucose metabolism (Yu et al. 2023). This surplus of reducing equivalents can enhance (co-)fermentation processes, especially for compounds more oxidized than glucose, such as acetic acid (Xiberras et al. 2019).

### Ethanol production

Bioethanol produced from renewable carbohydrates is a promising substitute or additive to petrol, helping reduce environmental pollution. First-generation bioethanol is derived from sugarcane, corn, or sugar beets. Significant efforts have been made to develop cost-effective second-generation ethanol from lignocellulosic biomass (Kurylenko et al. 2016, Ruchala et al. 2020). However, processing raw cellulosic material requires expensive pretreatment and enzymatic hydrolysis (Li et al. 2018a). Crude glycerol is a low-cost alternative feedstock that requires no such processing. Ethanol production from glycerol has been estimated to cost nearly 40% less than from corn-based sugars (Yazdani and Gonzalez 2007).

Since most microorganisms metabolize glycerol via respiration, few wild-type strains can ferment it to ethanol efficiently (Yazdani and Gonzalez 2007). Some researchers propose that glycerol metabolism via the G3P pathway is primarily respiratory, while the DHA pathway represents a more fermentative route. (Hong et al. 2010, Yu et al. 2010). Accordingly, the overexpression of genes encoding GDH and DHAK has mainly been pursued as a strategy to improve glycerol fermentation efficiency.

Ethanol is synthesized from pyruvate by pyruvate decarboxylase (PDC) and alcohol dehydrogenase (ADH) (Fig. 1). The expression levels of their corresponding genes significantly influence ethanol production (Nikel et al. 2010). *Saccharomyces cerevisiae*is highly efficient at ethanol production from sugars due to the effectiveness of its PDC and ADH enzymes, but its poor growth on glycerol limits ethanol production from this substrate.

To overcome this, several metabolic engineering strategies were applied to *S. cerevisiae*. Overexpressing GDH, DHAK, and the putative glycerol transporter Gup1 (its direct involvement in glycerol transport was later disproved) increased ethanol production 3.4-fold to 2.4 g/l (Yu et al. 2010). Subsequent deletions of *GPD2* and *FPS1* boosted production to 4.4 g/l. Additional overexpression of PDC and ADH genes further increased the ethanol titer to 5.4 g/l (Yu et al. 2012).

The methylotolerant, thermotolerant yeast *Ogataea polymorpha* (formerly *Hansenula polymorpha*) is more resistant to methanol and heavy metal impurities found in crude glycerol. In strain DL1 (now *O. parapolymorpha*), expression of *Zymomonas mobilis* genes *pdc* and *adhB* under a glyceraldehyde-3-phosphate dehydrogenase gene promoter led to production of 2.74 g/l ethanol, a 3.3-fold increase over the wild type. Coexpression of *dhaD* and *dhaKLM* from *Klebsiella pneumoniae* led to the accumulation of 3.1 g/l ethanol (Hong et al. 2010).

Overexpressing the native *ADH1* in a *Δadh1* DL1 background showed no improvement (Suwannarangsee et al. 2010). However, simultaneous overexpression of *PDC1* and *ADH1* in *O. polymorpha* NCYC495 increased ethanol production to 5.0 g/l at 45°C (Kata et al. 2016). Further enhancement through overexpression of genes involved in either G3P or DHA glycerol utilization pathways and *FPS1* from *K. phaffii* resulted in up to 10.7 g/l ethanol from pure glycerol. Still, only 3.6 g/l ethanol was achieved from crude glycerol, likely due to toxic impurities (Semkiv et al. 2019). Ethanol production could be further increased by simultaneously activating both the G3P and DHA pathways; however, this has not been done yet.

*Pachysolen tannophilus*, known for efficient xylose fermentation, has also shown promise for ethanol production from glycerol. Under aerobic conditions, it accumulated 4 g/l ethanol (Maleszka et al. 1982). Strain CBS4044 produced 17.5 g/l of ethanol from 5% crude glycerol and 28.1 g/l of ethanol during fed-batch fermentation (Liu et al. 2012). Immobilized *P. tannophilus* Y-475 cells in cryogels demonstrated ethanol yields up to 90% of theoretical maximum (Stepanov and Efremenko 2017).

Crude glycerol impurities (e.g. methanol, salts) did not adversely affect *P. tannophilus* ethanol production or viability (Liu et al. 2012). However, growth ceased at ethanol concentrations above 40 g/l (Zhao et al. 2010), indicating the need to improve its ethanol tolerance—potentially through adaptive evolution or UV mutagenesis (Watanabe et al. 2011). Overall, *P. tannophilus* is a robust candidate for bioethanol production from unrefined glycerol feedstocks.

Isopropanol, another widely used biofuel, can also be produced from crude glycerol. A recombinant *Y. lipolytica* strain engineered with the acetoacetyl-CoA synthase gene (*nphT7*) and additional isopropanol biosynthetic genes, along with an optimized glycerol-based medium, was employed for production (Fig. 1). In fermenter cultivation using pure glycerol, the maximum isopropanol titer reached 1.94 g/l. When crude glycerol was used as the substrate, 1.60 g/l of isopropanol was produced (Shi et al. 2022).

### Biodiesel (lipid production)

One of the most promising applications for crude glycerol is its use as a feedstock in the microbial production of lipids, often referred to as single-cell oils (SCO) (Khot et al. 2012). These microbial oils offer a sustainable alternative to plant-based oils traditionally used in biodiesel production, thus addressing concerns associated with land use and the food-versus-fuel debate. SCOs are synthesized by oleaginous microorganisms—including certain yeasts, bacteria, fungi, and microalgae—which can accumulate lipids up to 70% of their cell dry weight (CDW) (Saenge et al. 2011).

Oleaginous yeasts are especially attractive for lipid production due to their high lipid content, adaptability to various substrates, and ease of cultivation in bioreactors, independent of season or climate. Common lipid-accumulating yeasts include species from the genera *Yarrowia, Rhodotorula, Rhodosporidiobolus, Candida*, and *Lipomyces* (Lei et al. 2024). These yeasts typically store lipids as triacylglycerols (Fig. 1) within intracellular lipid bodies and produce fatty acid (FA) profiles similar to plant oils, making them suitable for biodiesel and oleochemical applications (Li et al. 2008).

However, commercial-scale microbial oil production remains cost-prohibitive, largely due to the price of carbon substrates (Koutinas et al. 2014). This has prompted interest in low-cost feedstocks, among which crude glycerol stands out for its abundance and low cost. Several oleaginous yeast species can metabolize glycerol efficiently, making it a viable substrate for SCO production.

The theoretical lipid yield from glycerol is ~0.3 g/g (Yang et al. 2014), though actual yields are lower and depend on various factors, including strain type, fermentation conditions, and the composition of crude glycerol.

A key parameter is the carbon-to-nitrogen (C/N) ratio. High C/N ratios favor lipid accumulation, while low ratios promote cell growth. For instance, *Lipomyces starkeyi* cultivated with a C/N ratio of 150 accumulated lipids up to 68% of its CDW, compared to only 40% at a C/N of 60 (Angerbauer et al. 2008).

The type of nitrogen source also impacts lipid synthesis. Some yeast strains prefer organic nitrogen sources like peptone or yeast extract, while others perform better with inorganic sources such as ammonium salts (Cheirsilp et al. 2011, Poli et al. 2014, Liu et al. 2016).

Additional factors that enhance lipid accumulation include controlled pH (Saenge et al. 2011, Chen et al. 2018, Manowattana et al. 2018), low oxygen levels (Yen and Zhang 2011, Manowattana et al. 2018), and the addition of salts or organic acids (Saenge et al. 2011, Manowattana et al. 2018).

Crude glycerol’s impurities—such as methanol, salts, and FA residues—can influence yeast performance. While certain impurities may enhance lipid production, others, especially methanol, often inhibit cell growth and lipid accumulation. However, the impact is strain-specific, and some yeasts tolerate or even thrive in the presence of these compounds. Strategies such as strain adaptation or fed-batch fermentation can mitigate inhibitory effects (Shen et al. 2011, Signori et al. 2016). Fed-batch systems, where glycerol is added gradually, have proven effective in avoiding substrate inhibition and enhancing yields. For example, one study achieved lipid productivity of 1 g/l/h using this approach (Koutinas et al. 2014). Compounds known to act as stress protectants in yeast cells can also help mitigate the negative effects of impurities in crude glycerol. For instance, l-proline enhanced the growth and lipid production of *Rhodotorula toruloides* cultured on crude glycerol, even in the presence of high concentrations of methanol and salt (Kamal et al. 2020).

Yeasts begin accumulating lipids during the growth phase, with maximum lipid content typically reached in early stationary phase (Beopoulos et al. 2008). To maximize yield, timely harvesting is crucial, as lipids can later be degraded into free FAs. This degradation can be suppressed in media limited in both nitrogen and magnesium (Bellou et al. 2016).

The FA profile of SCOs is critical for biodiesel quality (Pinzi et al. 2009). Lipids rich in monounsaturated FAs like oleic acid are ideal. Many yeasts naturally produce lipids high in oleic, palmitic, and linoleic acids. Interestingly, the use of crude glycerol can shift the FA profile, often increasing oleic or linoleic acid content depending on the yeast strain and cultivation conditions (Fakas et al. 2009, Spier et al. 2015, Signori et al. 2016).

The most studied oleaginous yeasts include *Y. lipolytica* (Papanikolaou and Aggelis 2002), *R. toruloides* (Shen et al. 2013), and *Rhodotorula glutinis* (Chi et al. 2011). *Yarrowia lipolytica*, in particular, is a model organism for lipid metabolism and can utilize various substrates, including crude glycerol (Beopoulos et al. 2009). For instance, the UFLA CM-Y9.4 strain accumulated 63.4% lipids (w/w) when grown on 30% crude glycerol (Souza et al. 2014). Another strain, SKY7, produced 43.8% lipids with 14.8 g/l biomass under optimized conditions (Kuttiraja et al. 2016). Derivatives of the *Y. lipolytica* strain MUCL 28849, developed through adaptive laboratory evolution, showed a 1.9–3.6-fold increase in dry biomass and a 1.1–1.6-fold increase in lipid concentration compared to the parental strain when cultivated in 15% (v/v) crude glycerol (Tsirigka et al. 2023). *Yarrowia lipolytica* strain JMY3580, constructed by overexpressing the *DGA2* gene (coding for diacylglycerol acyltransferase) in a Q4 strain background (*dga1Δ dga2Δ lro1Δ are1Δ*), exhibited restored triacylglycerol synthesis and achieved lipid accumulation exceeding 40% of biomass on a glycerol-based medium, with levels surpassing 50% under fed-batch conditions, significantly outperforming the wild-type strain and altering FA composition (Gajdos et al. 2017). In some cases, crude glycerol was coutilized with molasses (Rakicka et al. 2015) or palm oil mill effluent (Louhasakul and Cheirsilp 2013), further reducing substrate costs.

Despite these successes, *Y. lipolytica* typically accumulates lipids only up to 30% CDW without genetic or process optimization (Munch et al. 2015). Moreover, in the stationary phase, it may divert metabolism toward citric acid and other byproducts, reducing lipid content (Papanikolaou et al. 2013). Thus, other yeast species may be preferable when high lipid yields are desired without significant byproduct formation.

Several comparative studies have identified superior strains for lipid production from glycerol. *Lipomyces lipofer* NRRL Y-1155 achieved lipid content of 57.6% with crude glycerol (Spier et al. 2015). *Clavispora freyschussii* produced 28 g/l lipids at a productivity of 0.28 g/l/h in continuous culture (Raimondi et al. 2014). *Rhodosporidiobolus fluvialis* DMKU-RK253 reached 65.2% lipid content using crude glycerol in flask culture (Polburee et al. 2015). *Meyerozyma guilliermondii* BI281A was selected via Nile Red staining for its high lipid content with a favorable FA profile (Ramirez-Castrillon et al. 2017). *Pseudozyma* sp. TYC-2187 produced 15.7 g/l lipids in just 48 h on crude glycerol medium (Takakuwa et al. 2013). During fed-batch fermentation with 40 g/l crude glycerol, *Rhodotorula paludigena* CM33 accumulated 46.32 g/l of biomass with a lipid content of 37.65% of the CDW (Sriphuttha et al. 2023). *Rhodotorula toruloides*, particularly strain AS2.1389, has shown exceptional lipid accumulation (up to 74% CDW) with crude glycerol (Xu et al. 2012). Other notable strains include *Cutaneotrichosporon oleaginosus* (Signori et al. 2016), *Vanrija humicola*, and *W. anomalus* (Souza et al. 2017). These species demonstrate robust glycerol metabolism and favorable FA profiles for biodiesel.

Crude glycerol has also been effectively used alongside other waste substrates or hemicellulose hydrolysate. For example, adding a small amount of hemicellulose hydrolysate to crude glycerol enhanced glycerol metabolism in *R. toruloides* CBS 14 and *R. glutinis* CBS 3044, shortening fermentation by ∼24 hours. *Rhodotorula toruloides* showed a lipid yield of 0.25 g/g with the mixture versus 0.20 g/g on glycerol alone, while *R. glutinis* showed similar yields on both substrates (0.15–0.16 g/g) (Chmielarz et al. 2021). *Rhodotorula glutinis* utilized crude glycerol in combination with brewing waste (thin stillage) (Yen et al. 2012); *R. toruloides*—in combination with orange peel waste (Carota et al. 2020), while *C. oleaginosus* cofermented crude glycerol with corn steep liquor and yeast autolysate (Thiru et al. 2011). These integrated waste valorization approaches improve economic viability and sustainability by turning multiple industrial byproducts into value-added products.

In conclusion, crude glycerol is a viable, low-cost substrate for microbial lipid production. With careful strain selection, optimization of fermentation conditions, and impurity management, it is possible to achieve high lipid yields with favorable FA profiles suitable for biodiesel and other applications. Continued research into strain development, process engineering, and cosubstrate utilization will be key to scaling up SCO production for industrial use.

## Glycerol bioconversion to value-added products

### Organic acids production

Several yeast strains are capable of converting glycerol into valuable organic acids such as succinic acid, citric acid, isocitric acid, lactic acid, malic acid, and 5-aminolevulinic acid (Fig. 1). Notable examples include *S. cerevisiae* and *Y. lipolytica*, which have been metabolically engineered to enhance carbon flux toward these products. Through pathway optimization and transporter engineering, these yeasts have shown great potential for sustainable organic acid production from glycerol.

Succinic acid has broad range of applications across the food, chemical, and agricultural industries (Ahn et al. 2016). It serves as a key precursor for the synthesis of value-added compounds such as γ-butyrolactone, 1,4-butanediol, tetrahydrofuran, and other important chemical products (Mckinlay et al. 2007).

*Saccharomyces cerevisiae* was engineered for succinic acid production from glycerol and carbon dioxide (Fig. 1). The endogenous FAD-dependent G3P pathway for glycerol catabolism in *S. cerevisiae* was replaced by the synthetic NAD^+^-dependent DHA pathway (Xiberras et al. 2020). For that reason, NAD^+^-dependent GDH from *O. parapolymorpha*, endogenous *DAK1* and aquaglyceroporin Fps1 from *C. jadinii* to increase the glycerol uptake rate were overexpressed and integrated at the *GUT1* locus (Klein et al. 2016b).

To establish the cytosolic reductive succinic acid pathway from oxaloacetate to succinic acid, the following enzymes were overexpressed in the background of the previously engineered strain: the endogenous peroxisomal malate dehydrogenase (*MDH3*), responsible for the reduction of oxaloacetate to malate; the heterologous cytosolic fumarase (FumR) from *Rhizopus arrhizus*, catalyzing the conversion of malate to fumarate; and the peroxisomal fumarate reductase (FRDg) from *Trypanosoma brucei*, facilitating the reduction of fumarate to succinic acid. To redirect Mdh3 and Frd to the cytosol, their peroxisomal targeting signals were removed. With the additional expression of the heterologous dicarboxylic acid transporter DCT-02 from *Aspergillus niger*, a maximum succinic acid titer of 10.7 g/l and a yield of 0.22 g/g glycerol were achieved (Xiberras et al. 2020).

An optimized *S. cerevisiae* strain for succinic acid production was constructed through the additional overexpression of *PYC2*, an isozyme encoding pyruvate carboxylase. This enhancement improved the flux distribution from glycerol to succinic acid via the reverse tricarboxylic acid (TCA) pathway, promoted greater CO₂ fixation, and resulted in an increased succinic acid yield. The overexpression of *PYC2*, combined with increased availability of bicarbonate, the cosubstrate for the pyruvate carboxylase reaction, resulted in a maximum succinic acid yield of 0.60 g/g glycerol (Malubhoy et al. 2022).

To reduce the amount of carbon entering the CO₂-releasing oxidative TCA cycle, further optimization was performed by downregulating carbon flux through the oxidative TCA pathway. This was achieved by targeting the mitochondrial uptake of pyruvate and cytosolic intermediates of the reverse TCA pathway, as well as by disrupting the succinate dehydrogenase complex. The effects of deleting the genes *MPC1* and *MPC3* (subunits of the mitochondrial pyruvate carrier responsible for pyruvate uptake during growth on nonfermentable carbon sources), *OAC1* (a mitochondrial transporter that mediates oxaloacetate import by exchanging cytosolic oxaloacetate with mitochondrial sulfate), *DIC1* (a mitochondrial transporter facilitating the import of malate and succinic acid by exchanging them with mitochondrial phosphate), *SFC1* (a mitochondrial transporter primarily exchanging cytosolic succinic acid with mitochondrial fumarate), and *SDH1* (succinate dehydrogenase) on succinic acid production were investigated. The highest improvement was observed with the combined deletion of *MPC3* and *SDH1*. The engineered strain produced up to 45.5 g/l of succinic acid, achieved a maximum yield of 0.66 g/g glycerol, and accumulated the lowest levels of byproducts (Rendulić et al. 2024).

The aerobic yeast *Y. lipolytica* naturally produces organic acids such as citric acid, isocitric acid, and 2-oxoglutaric acid (Papanikolaou et al. 2002, Zhou et al. 2012). The *Y. lipolytica* strain PGC01003 was engineered by deleting the *SDH5* gene, which encodes subunit 5 of succinate dehydrogenase. This deletion blocks the conversion of succinic acid to fumaric acid in TCA, leading to the accumulation of succinic acid. Under optimized conditions, PGC01003 produced 43 g/l of succinic acid from crude glycerol. In fed-batch fermentation using a 2.5 l bioreactor, the strain achieved a succinic acid titer of 160 g/l (Gao et al. 2016).

However, a major drawback of this strain is its low growth rate when glycerol is used as the carbon source. To address this, the *GUT1* gene, which encodes GK, was overexpressed in strain PGC01003 to enhance glycerol uptake. The resulting strain exhibited a 13.5% increase in glycerol uptake compared to the parental strain. Overexpression of *GUT1* also had a positive impact on succinic acid production, with the titer, yield, and productivity in batch bioreactor cultivation increasing by 32%, 39%, and 143%, respectively, relative to PGC01003 (Ong et al. 2020).

Another limitation of strain PGC01003 was the accumulation acetic acid as a by-product, which hindered further improvements in succinic acid production. Additionally, the need for frequent pH adjustments increased downstream processing costs in industrial applications. The deletion of the *PDC* gene, which encodes PDC, along with the overexpression of endogenous acetyl-CoA synthetase from *Y. lipolytica* and a heterologous version from *Salmonella enterica*, did not reduce acetic acid formation. The deletion of the *ACH1* gene, which encodes acetyl-CoA hydrolase, inhibited the formation of acetic acid from acetyl-CoA, thereby reducing the reverse conversion of succinic acid to succinyl-CoA. This reduction in acetic acid concentration also enhanced cell growth, leading to improved succinic acid production (Cui et al. 2017). However, the formation of by-products, primarily pyruvate and its upstream metabolites, was observed. To channel pyruvate toward succinic acid production, the genes *PCK* (encoding phosphoenolpyruvate carboxykinase) from *S. cerevisiae* and *Y. lipolytica, PYC* (encoding pyruvate carboxylase) from *S. cerevisiae* and *Y. lipolytica*, as well as the endogenous *Y. lipolytica* genes *CIT* (citrate synthase), *ACO* (aconitase), *ICL* (isocitrate lyase), and *MLS* (malate synthase) were overexpressed. Overexpression of ScPCK completely eliminated pyruvate accumulation and resulted in a maximum succinic acid yield of 0.5 g/g. Overexpression of the endogenous *SCS2* gene, encoding the succinyl-CoA synthase beta subunit, further enhanced succinic acid production. In fed-batch fermentation, the final strain PGC202 produced 110.7 g/l of succinic acid with a yield of 0.53 g/g glycerol without pH control (Cui et al. 2017).

An optimized *in situ* fibrous bed bioreactor using sugarcane bagasse as immobilization material and crude glycerol as a substrate enabled *Y. lipolytica* to produce a highest titer of succinic acid reaching 209.7 g/l in fed-batch fermentation (Li et al. 2018b). Nitrogen limitation was also shown to reduce biomass accumulation and increase succinic acid yields in strains PGC01003 and PGC202 by 18% and 62%, respectively (Billerach et al. 2021).

Citric acid has broad applications across the food, beverage, pharmaceutical, chemical, and environmental industries. Citric acid is considered the most widely consumed organic acid across various industries (Latif et al. 2024).

*Yarrowia lipolytica* shows adaptable metabolism, producing mainly polyols at pH 2.5–3.5 and favoring citric acid synthesis at pH 4.5–7.5 (Egermeier et al. 2017). The cooverexpression of GK and GPD enhanced glycerol consumption, leading to increased production of citric acid and erythritol (Mirończuk et al. 2016).

An acetate-negative UV mutant of *Y. lipolytica* producing citrate (Rywińska et al. 2010) was metabolically engineered to enhance citric acid production. The genes *GUT1* (GK), *CIT1* (methylcitrate synthase), and *YALI0E34672g* (encoding the mitochondrial succinate–fumarate transporter) were overexpressed under the control of the strong, constitutive TEF promoter. The highest citric acid titer was achieved in the strain overexpressing *GUT1* (Rywińska et al. 2024).

Cex1 was identified as the first confirmed citrate exporter in *Y. lipolytica*, with its disruption abolishing citrate production and overexpression enhancing it 5.2-fold in a low-producing strain. Heterologous expression of Cex1 in *S. cerevisiae* and *A. niger* validated its citrate export function, providing a key tool for advancing metabolic engineering of citric acid secretion in yeasts (Erian et al. 2020).

Isocitric acid is of interest for organic synthesis and medical applications due to its biological activity (Heretsch et al. 2008). Under nitrogen starvation, *Y. lipolytica* produces a mixture of citric and isocitric acids, with their ratio largely influenced by the carbon source. High-level expression of the *ACO1* gene, which encodes aconitase, shifted the citric acid/isocitric acid production profile toward increased isocitric acid formation during cultivation on glycerol (Holz et al. 2009). Enhanced isocitric acid production in *Y. lipolytica* from glycerol was achieved by overexpressing either the *CIT1* or *CIT2* gene, both encoding citrate synthase (Hapeta et al. 2020). It has been shown that the mitochondrial succinate–fumarate carrier Sfc1 regulates isocitric acid efflux from the mitochondria. Overexpression of *SFC1* shifted the isocitric acid/citric acid ratio in favor of isocitric acid, resulting in the production of 43.3 g/l of isocitric acid during cultivation with glycerol (Yuzbasheva et al. 2021).

Lactic acid is a versatile organic acid with a broad range of applications. It is widely used in the food, pharmaceutical, textile, leather, and chemical industries, making it the most commercially significant hydroxycarboxylic acid globally (Díaz-Orozco et al. 2025). Additionally, lactic acid serves as the monomer for producing biodegradable polylactic acid, a major bioplastic that has attracted significant attention and is widely used in the automotive, packaging, and cosmetic industries (Huang et al. 2025). Although numerous studies have focused on engineering yeast strains for lactic acid production from conventional substrates (Tsaruk et al. 2025), the utilization of glycerol as a carbon source for lactate biosynthesis remains relatively uncommon.

The methylotrophic yeast *K. phaffii*, engineered to express bovine l-lactate dehydrogenase (LDH), produced lactic acid with a yield of 0.47 g/g of glycerol. Disruption of the gene encoding PDC increased the lactic acid yield to 0.65 g/g of glycerol, while simultaneously reducing acetic acid accumulation as a byproduct (Melo et al. 2018). Introduction of a newly identified endogenous lactate transporter to the strain expressing LDH further enhanced the yield, reaching 0.67 g/g of glycerol (de Lima et al. 2016).

Malic acid, an important four-carbon dicarboxylic acid, is broadly applied across the food, chemical, and pharmaceutical sectors (Wei et al. 2021). *Yarrowia lipolytica* demonstrated strong potential for malic acid production from glycerol. To enhance malic acid biosynthesis in this yeast species, key endogenous genes of the glyoxylate cycle—*ICLC* (isocitrate lyase), *MSE* (malate synthase), and *MDHE2* (malate dehydrogenase)—were overexpressed in conjunction with a heterologous malate transporter gene from *S. pombe*. The engineered strain produced 27 g/l of malic acid from glycerol, which was further increased to 37 g/l following medium optimization in shake flask cultures. Adaptive laboratory evolution enabled the engineered strain to tolerate lower pH conditions and accumulate up to 56 g/l of malic acid. Upon scale-up in a 5 l bioreactor, the malic acid titer reached 112.5 g/l (Wang et al. 2024).

5-Aminolevulinic acid is a naturally occurring, nontoxic precursor of tetrapyrroles such as chlorophyll, heme, and vitamin B12, synthesized via two biosynthetic pathways (C4 and C5) in various organisms (Kang et al. 2012). It serves important roles in medicine as a photosensitizer for tumor treatment, in livestock as a feed additive to enhance iron levels and immunity, and in plants to regulate growth and development (Jiang et al. 2022). Due to its diverse biological functions and environmental safety, 5-aminolevulinic acid has become a focus of increasing scientific research and biotechnological interest. The C4 pathway for 5-aminolevulinic acid biosynthesis, which utilizes succinyl-CoA and glycine as precursors, was enhanced by overexpressing the *HEM1* gene from *S. cerevisiae*, encoding 5-aminolevulinic acid synthase, in an SDH-deficient, succinic acid-producing *Y. lipolytica* strain. To implement the heterologous C5 pathway, which uses glutamate as a precursor, *StHemA* from *Salmonella typhimurium* and *EcHemL* from *Escherichia coli* were coexpressed in the same strain. This genetic modification led to a further increase in 5-aminolevulinic acid production. By optimizing culture conditions, the 5-aminolevulinic acid titer reached 2.2 g/l in fed-batch fermentation using glycerol as the sole carbon source. The engineered *Y. lipolytica* strains provide a robust platform for further genetic and metabolic modifications, holding significant potential for advancing the commercial production of 5-aminolevulinic acid (Cui et al. 2021).

### Polyols production

Polyols such as 1,2-propanediol, erythritol, arabitol, mannitol, and xylitol are high-value substances with wide-ranging applications in the food, pharmaceutical, chemical, and cosmetic industries. The biotechnological production of these compounds from glycerol by yeast (Fig. 1) presents a sustainable and economically attractive alternative to traditional chemical synthesis. Below, several examples of yeast-based processes for polyol production from glycerol are presented and discussed.

1,2-Propanediol is a chemical with high industrial demand. It is widely utilized across the building materials, chemical, and pharmaceutical sectors as a monomer for the production of polyester resins, antifreeze agents, liquid detergents, biofuels, cosmetics, food products (Tao et al. 2021). The engineering of a *S. cerevisiae* strain capable of producing 1,2-propanediol using glycerol as the carbon source has been described (Jung et al. 2011). The overexpression of *GUP1* (indirectly involved in glycerol uptake and metabolism) along with the G3P pathway genes *GUT1* and *GUT2*, combined with the heterologous expression of *gdh* from *Ogataea angusta*, enhanced glycerol assimilation in *S. cerevisiae*. Furthermore, the introduction of *mgsA* and *gldA* from *E. coli*, encoding methylglyoxal synthase and GDH respectively, enabled the conversion of DHAP to 1,2-propanediol, resulting in a production of 2.2 g/l of 1,2-propanediol (Jung et al. 2011). To fully utilize the reducing power of glycerol, cytosolic NAD(P)H must be conserved; however, in the G3P pathway, electrons from glycerol oxidation are transferred via FADH₂ to the mitochondrial respiratory chain, limiting the pool of cytosolic reducing equivalents (Klein et al. 2016a). To enhance cytosolic NAD⁺ utilization and improve 1,2-propanediol production, the native G3P pathway in *S. cerevisiae* was replaced with a synthetic NAD⁺-dependent DHA pathway. This approach involved deletion of the *GUT1* gene and expression of the *gdh* gene from *O. parapolymorpha*, encoding a NAD⁺-dependent GDH that catalyzes the oxidation of glycerol to DHA. Additionally, the native *DAK1* gene, encoding DHAK responsible for DHA phosphorylation, was overexpressed (Klein et al. 2016a). The resulting strain, further engineered with a heterologous methylglyoxal pathway and reduced TPI activity to boost DHAP availability, achieved the highest reported 1,2-propanediol titer in yeast to date, reaching 4.3 g/l with a yield of 129 mg/g of glycerol consumed (Islam et al. 2017).

Erythritol is a naturally occurring four-carbon sugar alcohol widely used as a low-calorie sweetener in food and beverage industries due to its zero glycemic index and noncariogenic properties. It offers about 70% of the sweetness of sucrose while being well-tolerated by the human body, making it suitable for diabetic and calorie-restricted diets. Beyond its role as a sweetener, erythritol also finds applications in pharmaceuticals and oral care products for its antioxidant and dental health-promoting effects (Li et al. 2024a). *Yarrowia lipolytica* has been recognized as the predominant host for erythritol production, as the majority of its strains naturally secrete erythritol when grown on glycerol (Rakicka-Pustułka et al. 2021, Ziuzia et al. 2023). UV mutagenesis of *Y. lipolytica* Wratislavia K1 yielded the MK1 mutant strain, which demonstrated significantly enhanced erythritol production with minimal by-product formation. In batch culture, MK1 produced up to 82.2 g/l erythritol with a yield of 0.55 g/g and a productivity of 0.84 g/l/h, while by-products remained below 5% of total metabolites (Mironczuk et al. 2015). This strain was used to scale up erythritol production from glycerol in a 500 l pilot-scale bioreactor. Erythritol production reached 180 g/l, with a productivity of 1.25 g/l/h and a yield of 0.53 g/g of crude glycerol (Rakicka-Pustułka et al. 2020). Metabolic engineering approaches were applied to further enhance erythritol production (Fan et al. 2025). The roles of seven key genes in erythritol biosynthesis were investigated in *Y. lipolytica*, with *TKL1* (transketolase) and *TAL1* (transaldolase) identified as major contributors to improved production. Cooverexpression of *GUT1, TPI1, TKL1*, and *TAL1*, along with deletion of *EYD1* (erythritol dehydrogenase), led to ~40 g/l erythritol from glycerol. Further overexpression of *RKI1* (ribose-5-phosphate isomerase) and *AMPD* (AMP deaminase) led to an increased erythritol production of up to 52 g/l (Zhang et al. 2021). Another study describes the selection of a strain capable of utilizing crude glycerol following UV mutagenesis. Based on the selected mutant, overexpression of *GUT1, GUT2*, and *TKL1*, along with the knockout of *EYD1*, led to the production of 150 g/l erythritol in a 5 l bioreactor, achieving a yield of 0.62 g/g and a productivity of 1.25 g/l/h from crude glycerol (Yang et al. 2022). In other study, deletion of *EYD1*, combined with the overexpression of endogenous *GUT1, TKL1, TAL1*, erythrose reductase, and *FPS1* from *S. cerevisiae*, led to the development of the ERY8 strain, which produced 176.66 g/l erythritol in a 5 l bioreactor from glycerol, with a yield of 0.631 g/g and a productivity of 1.23 g/l/h (Huang et al. 2023).

The erythrose reductase gene (YALI0B07117g) was identified as a key gene influencing erythritol biosynthesis in *Y. lipolytica*. Deletion of this gene redirected the carbon flux toward mannitol and arabitol, while its overexpression increased erythritol production from 41.15 to 59.83 g/l and improved productivity from 0.28 to 0.41 g/l/h using glycerol as the carbon source (Szczepańczyk et al. 2021). In another study, a mutant form of erythrose reductase (D46A), exhibiting 1.6-fold higher catalytic activity compared to the wild-type, was identified through whole-genome sequencing of a strain isolated via biosensor-guided adaptive evolution screening. This activity was further enhanced through combinatorial mutagenesis, achieving a 4.1-fold increase. Following further metabolic reconfiguration, the engineered strain G31 achieved an erythritol titer of 220.5 g/l with a productivity of 1.8 g/l/h in a 5 l bioreactor (Li et al. 2024b).

Arabitol, a five-carbon polyol, is gaining interest as a low-calorie, noncariogenic sweetener with potential applications in the food, oral healthcare, and pharmaceutical industries (Kordowska-Wiater 2015). *Debaryomyces* sp. has been reported to produce arabitol from glycerol, achieving a titer of 47.5 g/l with a yield of 0.63 g/g (Filippousi et al. 2019). *Kurtzmaniella quercitrusa* demonstrated high arabitol production reaching 85.1 g/l from crude glycerol after 10 days with a yield of 0.40 g/g (Yoshikawa et al. 2014b). The yeast *W. anomalus* is known for its ability to produce arabitol when cultivated in a glycerol-based, nitrogen-limited medium. Fed-batch fermentation was optimized using central composite design. Increased cell density, constant glycerol feeding, and temperature adjustment led to a titer of 265 g/l of arabitol, with a conversion yield of 0.74 g/g and productivity of 0.82 g/l/h (Raimondi et al. 2022). *Wickerhamomyces anomalus* has proven to be a promising arabitol producer from glycerol, showing strong potential for application in immobilized cell processes due to its sustained viability and arabitol production independent of growth (Ranieri et al. 2024).

Mannitol is a naturally occurring six-carbon sugar alcohol widely used in the food and pharmaceutical industries due to its beneficial properties, including its role as a natural sweetener with low metabolic impact and a zero glycemic index (Chen et al. 2020). Screening of yeast strains isolated from environmental samples for mannitol production from crude glycerol identified *Candida azyma* as the most efficient producer, achieving 31.8 g/l in flask cultures. In bioreactor cultivation, *C. azyma* produced 50.8 g/l of mannitol with a yield of 0.30 g/g of glycerol (Yoshikawa et al. 2014a). Screening of yeasts from the *Yarrowia* clade identified *Yarrowia divulgata* and *Yarrowia oslonensis* as particularly efficient polyol producers. Both species generated high levels of polyols from soap-derived glycerol (59.8–62.7 g/l) and biodiesel-derived crude glycerol (76.8–79.5 g/l). In bioreactor cultures, *Y. divulgata* and *Y. oslonensis* produced mannitol with yields of 0.25 g/g and 0.30 g/g, and productivities of 0.43 g/l/h and 0.60 g/l/h, respectively (Rakicka-Pustułka et al. 2021). In another studies *Y. lipolytica* strains were screened for their ability to biosynthesize mannitol from glycerol. The S3 strain produced mannitol as the dominant polyol, accounting for up to 70% of total polyols, and showed high resistance to NaCl and osmotic stress. Implementation of a fed-batch strategy with 200 g/l glycerol nearly doubled mannitol production, increasing from 44.6–75.9 g/l (Juszczyk et al. 2023).

Xylitol is a value-added chemical widely used in food, pharmaceuticals, and oral care products due to its sweetening properties and health benefits. Glycerol has emerged as an effective cosubstrate for xylitol biosynthesis using various yeast species. *Kluyveromyces marxianus* utilizes glycerol via the G3P pathway, enhancing NADPH regeneration and supporting high xylitol yields (up to 322.07 g/l), particularly when engineered with CRISPR/Cas9 and fed industrial by-products (Ren et al. 2022). Similarly, *Y. lipolytica*, grown on glycerol–xylose mixtures, achieved up to 53.2 g/l xylitol with yields approaching 0.97 g/g, and maintained efficiency with crude glycerol and lignocellulosic hydrolysates (Prabhu et al. 2020). In *S. cerevisiae*, overexpression of the endogenous aldose reductase gene *GRE3* and the xylose transporter gene *SUT1* enabled enhanced xylitol production from hemicellulosic hydrolysate, achieving a productivity of 318.6 mg/l/h when glycerol was used as a cosubstrate. Notably, the glycerol-to-xylitol conversion was more efficient (0.47 mol/mol) than the glucose-based conversion (approximately 0.04 mol/mol, corresponding to ~23.7 mol glucose consumed per mole of xylitol) (Kogje and Ghosalkar 2017). Such strategies support the development of cost-effective and sustainable bioprocesses for industrial xylitol production.

## Expanding the bioproduct potential of glycerol

Glycerol is widely regarded as a cheap and abundant substrate suitable for various bioconversion processes, primarily leading to the production of biofuels (such as ethanol and single cell oils), polyols, and organic acids. In addition to these applications, several studies have reported the potential of glycerol as a feedstock for the biosynthesis of high-value chemicals by yeasts. For instance, mutant strains of *Candida famata* have been shown to overproduce riboflavin from glycerol (Fedorovch and Sibirny, unpublished), which is of industrial relevance given that the more expensive glucose is currently the primary substrate used in commercial riboflavin production. It can be assumed that glycerol may serve as an efficient substrate for the production of the promising bacterial antibiotics roseoflavin and 8-aminoriboflavin in recombinant strains of the yeasts *K. phaffii* and *C. famata*. These antibiotics exhibit both antibacterial and antiplasmodial activities (Dmytruk et al. 2023, Hemasa et al. 2022).

The range of high-value chemicals that can be synthesized by yeasts from glycerol is expected to expand. For example, various *Rhodotorula* species are capable of accumulating carotenoids (Sriphuttha et al. 2023, Keskin et al. 2023), *Y. lipolytica* produces enantiopure (R)-1,2-octanediol (Godase et al. 2023), and recombinant laccase production on glycerol as a substrate has been demonstrated in *K. phaffii* (Pezzella et al. 2017). The two-stage glycerol feeding method increased the specific growth rate of *K. phaffii* during the preinduction phase for human granulocyte colony-stimulating factor production (Bahrami et al. 2009). These developments show promising potential and are likely to be pursued further.

However, the long-term availability of glycerol as a low-cost and abundant feedstock is closely tied to the future of the biodiesel industry. If the production of diesel vehicles is gradually phased out, biodiesel manufacturing—and consequently, crude glycerol generation—may decline significantly. Moreover, a shift from conventional biodiesel based on fatty acid methyl esters to hydrotreated vegetable oil (HVO) would further reduce glycerol availability, as HVO production does not generate glycerol as a byproduct. While this outcome appears plausible, it is not inevitable, and reliable forecasts remain uncertain at this stage.

## References

[bib1] Adler L, Blomberg A, Nilsson A. Glycerol metabolism and osmoregulation in the salt-tolerant yeast *Debaryomyces hansenii*. J Bacteriol. 1985;162:300–6. 10.1128/jb.162.1.300-306.1985. 3980438 PMC218989

[bib2] Ahmadpour D, Geijer C, Tamas MJ et al. Yeast reveals unexpected roles and regulatory features of aquaporins and aquaglyceroporins. Biochim Biophys Acta. 2014;1840:1482–91. 10.1016/j.bbagen.2013.09.027. 24076236

[bib3] Ahn JH, Jang YS, Lee SY. Production of succinic acid by metabolically engineered microorganisms. Curr Opin Biotechnol. 2016;42:54–66. 10.1016/j.copbio.2016.02.034. 26990278

[bib4] Albertyn J, Hohmann S, Thevelein JM et al. GPD1, which encodes glycerol-3-phosphate dehydrogenase, is essential for growth under osmotic stress in *Saccharomyces cerevisiae*, and its expression is regulated by the high-osmolarity glycerol response pathway. Mol Cell Biol. 1994;14:4135–44. 8196651 10.1128/mcb.14.6.4135-4144.1994 PMC358779

[bib5] Ali O, Szabó A. Review of eukaryote cellular membrane lipid composition, with special attention to the fatty acids. Int J Mol Sci. 2023;24:15693. 10.3390/ijms242115693. 37958678 PMC10649022

[bib6] Angerbauer C, Siebenhofer M, Mittelbach M et al. Conversion of sewage sludge into lipids by *Lipomyces starkeyi* for biodiesel production. Bioresour Technol. 2008;99:3051–6. 10.1016/j.biortech.2007.06.045. 17719773

[bib7] Ansell R, Granath K, Hohmann S et al. The two isoenzymes for yeast NAD+-dependent glycerol 3-phosphate dehydrogenase encoded by GPD1 and GPD2 have distinct roles in osmoadaptation and redox regulation. EMBO J. 1997;16:2179–87. 10.1093/emboj/16.9.2179. 9171333 PMC1169820

[bib8] Bahrami A, Shojaosadati SA, Khalilzadeh R et al. Prevention of human granulocyte colony-stimulating factor protein aggregation in recombinant *Pichia pastoris* fed-batch fermentation using additives. Biotechnol Appl Biochem. 2009;52:141–8. 10.1042/BA20070267. 18479251

[bib9] Baltanas R, Bush A, Couto A et al. Pheromone-induced morphogenesis improves osmoadaptation capacity by activating the HOG MAPK pathway. Sci Signal. 2013;6:ra26. 10.1126/scisignal.2003312. 23612707 PMC3701258

[bib10] Bellou S, Triantaphyllidou IE, Mizerakis P et al. High lipid accumulation in *Yarrowia lipolytica* cultivated under double limitation of nitrogen and magnesium. J Biotechnol. 2016;234:116–26. 10.1016/j.jbiotec.2016.08.001. 27498313

[bib11] Beopoulos A, Chardot T, Nicaud JM. *Yarrowia lipolytica*: a model and a tool to understand the mechanisms implicated in lipid accumulation. Biochimie. 2009;91:692–6. 10.1016/j.biochi.2009.02.004. 19248816

[bib12] Beopoulos A, Mrozova Z, Thevenieau F et al. Control of lipid accumulation in the yeast *Yarrowia lipolytica*. Appl Environ Microbiol. 2008;74:7779–89. 10.1128/AEM.01412-08. 18952867 PMC2607157

[bib13] Billerach G, Preziosi-Belloy L, Lin CSK et al. Impact of nitrogen deficiency on succinic acid production by engineered strains of *Yarrowia lipolytica*. J Biotechnol. 2021;336:30–40. 10.1016/j.jbiotec.2021.06.001. 34090952

[bib14] Bjorkqvist S, Ansell R, Adler L et al. Physiological response to anaerobicity of glycerol-3-phosphate dehydrogenase mutants of *Saccharomyces cerevisiae*. Appl Environ Microbiol. 1997;63:128–32. 10.1128/aem.63.1.128-132.1997. 8979347 PMC168310

[bib15] Carly F, Vandermies M, Telek S et al. Enhancing erythritol productivity in *Yarrowia lipolytica* using metabolic engineering. Metab Eng. 2017;42:19–24. 10.1016/j.ymben.2017.05.002. 28545807

[bib16] Carota E, Petruccioli M, D’Annibale A et al. Mixed glycerol and orange peel-based substrate for fed-batch microbial biodiesel production. Heliyon. 2020;6:e04801. 10.1016/j.heliyon.2020.e04801. 32984573 PMC7494470

[bib17] Cheirsilp B, Suwannarat W, Niyomdecha R. Mixed culture of oleaginous yeast *Rhodotorula glutinis* and microalga *Chlorella vulgaris* for lipid production from industrial wastes and its use as biodiesel feedstock. New Biotechnol. 2011;28:362–8. 10.1016/j.nbt.2011.01.004. 21255692

[bib18] Chen J, Zhang X, Drogui P et al. The pH-based fed-batch for lipid production from *Trichosporon oleaginosus* with crude glycerol. Bioresour Technol. 2018;259:237–43. 10.1016/j.biortech.2018.03.045. 29567595

[bib19] Chen M, Zhang W, Wu H et al. Mannitol: physiological functionalities, determination methods, biotechnological production, and applications. Appl Microbiol Biotechnol. 2020;104:6941–51. 10.1007/s00253-020-10757-y. 32601737

[bib20] Chi Z, Zheng Y, Jiang A et al. Lipid production by culturing oleaginous yeast and algae with food waste and municipal wastewater in an integrated process. Appl Biochem Biotechnol. 2011;165:442–53. 10.1007/s12010-011-9263-6. 21567213

[bib21] Chilakamarry CR, Sakinah AMM, Zularisam AW. et al. Glycerol waste to value added products and its potential applications. Syst Microbiol Biomanuf. 2021;1:378–96. 10.1007/s43393-021-00036-w. 38624889 PMC8182736

[bib22] Chmielarz M, Blomqvist J, Sampels S et al. Microbial lipid production from crude glycerol and hemicellulosic hydrolysate with oleaginous yeasts. Biotechnol Biofuels. 2021;14:65. 10.1186/s13068-021-01916-y. 33712047 PMC7953724

[bib23] Cui Z, Gao C, Li J et al. Engineering of unconventional yeast *Yarrowia lipolytica* for efficient succinic acid production from glycerol at low pH. Metab Eng. 2017;42:126–33. 10.1016/j.ymben.2017.06.007. 28627452

[bib24] Cui Z, Zhu Z, Zhang J et al. Efficient 5-aminolevulinic acid production through reconstructing the metabolic pathway in SDH-deficient *Yarrowia lipolytica*. Biochem Eng J. 2021;174:108125. 10.1016/j.bej.2021.108125.

[bib25] de Lima PB, Mulder KCL, Melo NTM et al. Novel homologous lactate transporter improves L-lactic acid production from glycerol in recombinant strains of *Pichia pastoris*. Microb Cell Fact. 2016;15:158. 10.1186/s12934-016-0557-9. 27634467 PMC5025603

[bib26] de Nadal E, Posas F. The HOG pathway and the regulation of osmoadaptive responses in yeast. FEMS Yeast Res. 2022;22:foac013. 10.1093/femsyr/foac013. 35254447 PMC8953452

[bib27] Díaz-Orozco L, Moscosa Santillán M, Delgado Portales RE et al. Advances in L-lactic acid production from lignocellulose using genetically modified microbial systems. Polymers. 2025;17:322. 10.3390/polym17030322. 39940524 PMC11820014

[bib28] Dmytruk K, Ruchala J, Fayura L et al. Efficient production of bacterial antibiotics aminoriboflavin and roseoflavin in eukaryotic microorganisms, yeasts. Microb Cell Fact. 2023;22:132. 10.1186/s12934-023-02129-8. 37474952 PMC10357625

[bib29] Dujon B, Sherman D, Fischer G et al. Genome evolution in yeasts. Nature. 2004;430:35–44. 10.1038/nature02579. 15229592

[bib30] Egermeier M, Russmayer H, Sauer M et al. Metabolic flexibility of *Yarrowia lipolytica* growing on glycerol. Front Microbiol. 2017;8:49. 10.3389/fmicb.2017.00049. 28174563 PMC5258708

[bib31] Erian AM, Egermeier M, Rassinger A et al. Identification of the citrate exporter Cex1 of *Yarrowia lipolytica*. FEMS Yeast Res. 2020;20:foaa055. 10.1093/femsyr/foaa055. 32990722

[bib32] Eriksson P, Andre L, Ansell R et al. Cloning and characterization of GPD2, a second gene encoding sn-glycerol 3-phosphate dehydrogenase (NAD+) in *Saccharomyces cerevisiae*, and its comparison with GPD1. Mol Microbiol. 1995;17:95–107. 10.1111/j.1365-2958.1995.mmi_17010095.x. 7476212

[bib33] Fakas S, Papanikolaou S, Batsos A et al. Evaluating renewable carbon sources as substrates for single cell oil production by *Cunninghamella echinulata* and *Mortierella isabellina*. Biomass Bioenergy. 2009;33:573–80. 10.1016/j.biombioe.2008.09.006.

[bib34] Fan B, Liang X, Li Y et al. Biosynthesis and metabolic engineering of natural sweeteners. AMB Expr. 2025;15:50. 10.1186/s13568-025-01864-y. PMC11920521 40100508

[bib35] Farouk SM, Tayeb AM, Abdel-Hamid SMS et al. Recent advances in transesterification for sustainable biodiesel production, challenges, and prospects: a comprehensive review. Environ Sci Pollut Res Int. 2024;31:12722–47. 10.1007/s11356-024-32027-4. 38253825 PMC10881653

[bib36] Ferreira C, van Voorst F, Martins A et al. A member of the sugar transporter family, Stl1p is the glycerol/H+ symporter in *Saccharomyces cerevisiae*. Mol Biol Cell. 2005;16:2068–76. 10.1091/mbc.e04-10-0884. 15703210 PMC1073684

[bib37] Filippousi R, Antoniou D, Tryfinopoulou P et al. Isolation, identification and screening of yeasts towards their ability to assimilate biodiesel-derived crude glycerol: microbial production of polyols, endopolysaccharides and lipid. J Appl Microbiol. 2019;127:1080–100. 10.1111/jam.14373. 31286622

[bib38] Gajdos P, Nicaud JM, Certik M. Glycerol conversion into a single cell oil by engineered *Yarrowia lipolytica*. Eng Life Sci. 2017;17:325–32. 10.1002/elsc.201600065. 32624778 PMC6999298

[bib39] Gao C, Yang X, Wang H et al. Robust succinic acid production from crude glycerol using engineered *Yarrowia lipolytica*. Biotechnol Biofuels. 2016;9:179. 10.1186/s13068-016-0597-8. 27579143 PMC5004273

[bib40] Garlapati VK, Shankar U, Budhiraja A. Bioconversion technologies of crude glycerol to value added industrial products. Biotechnol Rep. 2016;9:9–14. 10.1016/j.btre.2015.11.002. PMC5360980 28352587

[bib41] Godase VP, Kumar VR, Kumar AR et al. Potential of *Y. lipolytica* epoxide hydrolase for efficient production of enantiopure (R)-1,2-octanediol. AMB Expr. 2023;13:77. 10.1186/s13568-023-01584-1. PMC10371975 37495892

[bib42] Hapeta P, Rakicka-Pustułka M, Juszczyk P et al. Overexpression of citrate synthase increases isocitric acid biosynthesis in the yeast *Yarrowia lipolytica*. Sustainability. 2020;12:7364. 10.3390/su12187364.

[bib43] Hemasa A, Mack M, Saliba K. Roseoflavin, a natural riboflavin analogue, possesses *in vitro* and *in vivo* antiplasmodial activity. Antimicrob Agents Chemother. 2022;66:e0054022. 10.1128/aac.00540-22. 36094195 PMC9578400

[bib44] Heretsch P, Thomas F, Aurich A et al. Syntheses with a chiral building block from the citric acid cycle: (2R,3S)-isocitric acid by fermentation of sunflower oil. Angew Chem Int Ed. 2008;47:1958–60. 10.1002/anie.200705000. 18236482

[bib45] Holz M, Förster A, Mauersberger S et al. Aconitase overexpression changes the product ratio of citric acid production by *Yarrowia lipolytica*. Appl Microbiol Biotechnol. 2009;81:1087–96. 10.1007/s00253-008-1725-6. 18850095

[bib46] Hong WK, Kim CH, Heo SY et al. Enhanced production of ethanol from glycerol by engineered *Hansenula polymorpha* expressing pyruvate decarboxylase and aldehyde dehydrogenase genes from *Zymomonas mobilis*. Biotechnol Lett. 2010;32:1077–82. 10.1007/s10529-010-0259-z. 20354759

[bib47] Huang L, Xiao B, Wang W et al. Multiplex modification of *Yarrowia lipolytica* for enhanced erythritol biosynthesis from glycerol through modularized metabolic engineering. Bioprocess Biosyst Eng. 2023;46:1351–63. 10.1007/s00449-023-02906-0. 37468580

[bib48] Huang S, Dong Q, Che S et al. Bioplastics and biodegradable plastics: a review of recent advances, feasibility and cleaner production. Sci Total Environ. 2025;969:178911. 10.1016/j.scitotenv.2025.178911. 40022973

[bib49] IndexBox . Crude glycerol. Global market report. 2024. https://www.indexbox.io/blog/crude-glycerol-world-market-overview-2024. Accessed July 7, 2025.

[bib50] Islam ZU, Klein M, Asskamp MR et al. A modular metabolic engineering approach for the production of 1,2-propanediol from glycerol by *Saccharomyces cerevisiae*. Metab Eng. 2017;44:223–35. 10.1016/j.ymben.2017.10.002. 29024819

[bib51] Jagtap SS, Bedekar AA, Singh V et al. Metabolic engineering of the oleaginous yeast *Yarrowia lipolytica* PO1f for production of erythritol from glycerol. Biotechnol Biofuels. 2021;14:188. 10.1186/s13068-021-02039-0. 34563235 PMC8466642

[bib52] Jiang M, Hong K, Mao Y et al. Natural 5-aminolevulinic acid: sources, biosynthesis, detection and applications. Front Bioeng Biotechnol. 2022;10:841443. 10.3389/fbioe.2022.841443. 35284403 PMC8913508

[bib53] Jung JY, Yun HS, Lee J et al. Production of 1,2-propanediol from glycerol in *Saccharomyces cerevisiae*. J Microbiol Biotechnol. 2011;21:846–53. 10.4014/jmb.1103.03009. 21876375

[bib54] Juszczyk P, Rywińska A, Kosicka J et al. Sugar alcohol sweetener production by *Yarrowia lipolytica* grown in media containing glycerol. Molecules. 2023;28:6594. 10.3390/molecules28186594. 37764370 PMC10534813

[bib55] Kamal R, Liu Y, Li Q et al. Exogenous l-proline improved *Rhodosporidium toruloides* lipid production on crude glycerol. Biotechnol Biofuels. 2020;13:159. 10.1186/s13068-020-01798-6. 32944075 PMC7490893

[bib56] Kang Z, Zhang J, Zhou J et al. Recent advances in microbial production of δ-aminolevulinic acid and vitamin B12. Biotechnol Adv. 2012;30:1533–42. 10.1016/j.biotechadv.2012.04.003. 22537876

[bib57] Kata I, Semkiv MV, Ruchala J et al. Overexpression of the genes PDC1 and ADH1 activates glycerol conversion to ethanol in the thermotolerant yeast *Ogataea* (*Hansenula*) *polymorpha*. Yeast. 2016;33:471–8. 10.1002/yea.3175. 27256876

[bib58] Keskin A, Ünlü AE, Takaç S et al. Utilization of olive mill wastewater for selective production of lipids and carotenoids by *Rhodotorula glutinis*. Appl Microbiol Biotechnol. 2023;107:4973–85. 10.1007/s00253-023-12625-x. 37329489

[bib59] Khot M, Kamat S, Zinjarde S et al. Single cell oil of oleaginous fungi from the tropical mangrove wetlands as a potential feedstock for biodiesel. Microb Cell Fact. 2012;11:71. 10.1186/1475-2859-11-71. 22646719 PMC3442963

[bib60] Klein M, Carrillo M, Xiberras J et al. Towards the exploitation of glycerol’s high reducing power in *Saccharomyces cerevisiae*-based bioprocesses. Metab Eng. 2016a;38:464–72. 10.1016/j.ymben.2016.10.008. 27750033

[bib61] Klein M, Islam ZU, Knudsen PB et al. The expression of glycerol facilitators from various yeast species improves growth on glycerol of *Saccharomyces cerevisiae*. Metab Eng Commun. 2016b;3:252–7. 10.1016/j.meteno.2016.09.001. 29468128 PMC5779717

[bib62] Kogje AB, Ghosalkar A. Xylitol production by genetically modified industrial strain of *Saccharomyces cerevisiae* using glycerol as co-substrate. J Ind Microbiol Biotechnol. 2017;44:961–71. 10.1007/s10295-017-1914-3. 28188449

[bib63] Kordowska-Wiater M. Production of arabitol by yeasts: current status and future prospects. J Appl Microbiol. 2015;119:303–14. 10.1111/jam.12807. 25809659

[bib64] Koutinas AA, Chatzifragkou A, Kopsahelis N et al. Design and techno-economic evaluation of microbial oil production as a renewable resource for biodiesel and oleochemical production. Fuel. 2014;116:566–77. 10.1016/j.fuel.2013.08.045.

[bib65] Kurylenko O, Semkiv M, Ruchala J et al. New approaches for improving the production of the 1st and 2nd generation ethanol by yeast. Acta Biochim Pol. 2016;63:31–8. 26619255 10.18388/abp.2015_1156

[bib66] Kuttiraja M, Douha A, Valero JR et al. Elucidating the effect of glycerol concentration and C/N ratio on lipid production using *Yarrowia lipolytica* SKY7. Appl Biochem Biotechnol. 2016;180:1586–600. 10.1007/s12010-016-2189-2. 27422535

[bib67] Lages F, Lucas C. Characterization of a glycerol/H+ symport in the halotolerant yeast *Pichia sorbitophila*. Yeast. 1995;11:111–9. 10.1002/yea.320110203. 7732721

[bib68] Lages F, Silva-Graca M, Lucas C. Active glycerol uptake is a mechanism underlying halotolerance in yeasts: a study of 42 species. Microbiology. 1999;145:2577–85. 10.1099/00221287-145-9-2577. 10517611

[bib69] Larsson C, Påhlman I-L, Ansell R et al. The importance of the glycerol 3-phosphate shuttle during aerobic growth of *Saccharomyces cerevisiae*. Yeast. 1998;14:347–57. 10.1002/(SICI)1097-0061(19980315)14:4<347::AID-YEA226>3.0.CO;2-9. 9559543

[bib70] Latif A, Hassan N, Ali H et al. An overview of key industrial product citric acid production by *Aspergillus niger* and its application. J Ind Microbiol Biotechnol. 2024;52:kuaf007. 10.1093/jimb/kuaf007. 40156584 PMC11956825

[bib71] Lee DH, Kim MD, Ryu YW et al. Cloning and characterization of CmGPD1, the *Candida magnoliae* homologue of glycerol-3-phosphate dehydrogenase. FEMS Yeast Res. 2008;8:1324–33. 10.1111/j.1567-1364.2008.00446.x. 19054133

[bib72] Lee J, Reiter W, Dohnal I et al. MAPK Hog1 closes the *S. cerevisiae* glycerol channel Fps1 by phosphorylating and displacing its positive regulators. Genes Dev. 2013;27:2590–601. 10.1101/gad.229310.113. 24298058 PMC3861672

[bib73] Lei Y, Wang X, Sun S et al. A review of lipid accumulation by oleaginous yeasts: culture mode. Sci Total Environ. 2024;919:170385. 10.1016/j.scitotenv.2024.170385. 38364585

[bib74] Levin DE. Regulation of cell wall biogenesis in *Saccharomyces cerevisiae*: the cell wall integrity signaling pathway. Genetics. 2011;189:1145–75. 10.1534/genetics.111.128264. 22174182 PMC3241422

[bib75] Li C, Gao S, Yang X et al. Green and sustainable succinic acid production from crude glycerol by engineered *Yarrowia lipolytica* via agricultural residue based in situ fibrous bed bioreactor. Bioresour Technol. 2018a;249:612–19. 10.1016/j.biortech.2017.10.011. 29091845

[bib76] Li J, Lu M, Guo X et al. Insights into the improvement of alkaline hydrogen peroxide (AHP) pretreatment on the enzymatic hydrolysis of corn stover: chemical and microstructural analyses. Bioresour Technol. 2018b;265:1–7. 10.1016/j.biortech.2018.05.082. 29860078

[bib77] Li L, Zhang Q, Shi R et al. Multidimensional combinatorial screening for high-level production of erythritol in *Yarrowia lipolytica*. Bioresour Technol. 2024a;406:131035. 10.1016/j.biortech.2024.131035. 38925409

[bib78] Li M, Ni Z, Li Z et al. Research progress on biosynthesis of erythritol and multi-dimensional optimization of production strategies. World J Microbiol Biotechnol. 2024b;40:240. 10.1007/s11274-024-04043-6. 38867081

[bib79] Li Q, Du W, Liu D. Perspectives of microbial oils for biodiesel production. Appl Microbiol Biotechnol. 2008;80:749–56. 10.1007/s00253-008-1625-9. 18690426

[bib80] Liu LP, Hu Y, Lou WY et al. Use of crude glycerol as sole carbon source for microbial lipid production by oleaginous yeasts. Appl Biochem Biotechnol. 2016;182:495–510. 10.1007/s12010-016-2340-0. 27988854

[bib81] Liu X, Jensen PR, Workman M. Bioconversion of crude glycerol feedstocks into ethanol by *Pachysolen tannophilus*. Bioresour Technol. 2012;104:579–86. 10.1016/j.biortech.2011.10.065. 22093973

[bib82] Liu X, Mortensen UH, Workman M. Expression and functional studies of genes involved in transport and metabolism of glycerol in *Pachysolen tannophilus*. Microb Cell Fact. 2013;12:27. 10.1186/1475-2859-12-27. 23514356 PMC3610204

[bib83] Louhasakul Y, Cheirsilp B. Industrial waste utilization for low-cost production of raw material oil through microbial fermentation. Appl Biochem Biotechnol. 2013;169:110–22. 10.1007/s12010-012-9965-4. 23151967

[bib84] Lucas C, Da Costa M, Van Uden N. Osmoregulatory active sodium-glycerol co-transport in the halotolerant yeast *Debaryomyces hansenii*. Yeast. 1990;6:187–91. 10.1002/yea.320060303.

[bib85] Luyten K, Albertyn J, Skibbe WF et al. Fps1, a yeast member of the MIP family of channel proteins, is a facilitator for glycerol uptake and efflux and is inactive under osmotic stress. EMBO J. 1995;14:1360–71. 10.1002/j.1460-2075.1995.tb07122.x. 7729414 PMC398221

[bib86] Maleszka R, Wang P, Schneider H. Ethanol production from D-galactose and glycerol by *Pachysolen tannophilus*. Enzyme Microb Technol. 1982;4:349–52. 10.1016/0141-0229(82)90059-X.

[bib87] Malubhoy Z, Bahia FM, de Valk SC et al. Carbon dioxide fixation via production of succinic acid from glycerol in engineered *Saccharomyces cerevisiae*. Microb Cell Fact. 2022;21:102. 10.1186/s12934-022-01817-1. 35643577 PMC9148483

[bib88] Manowattana A, Techapun C, Watanabe M et al. Bioconversion of biodiesel-derived crude glycerol into lipids and carotenoids by an oleaginous red yeast *Sporidiobolus pararoseus* KM281507 in an airlift bioreactor. J Biosci Bioeng. 2018;125:59–66. 10.1016/j.jbiosc.2017.07.014. 28827048

[bib89] Matsuzawa T, Ohashi T, Hosomi A et al. The gld1+ gene encoding glycerol dehydrogenase is required for glycerol metabolism in *Schizosaccharomyces pombe*. Appl Microbiol Biotechnol. 2010;87:715–27. 10.1007/s00253-010-2586-3. 20396879

[bib90] Mattanovich D, Graf A, Stadlmann J et al. Genome, secretome and glucose transport highlight unique features of the protein production host *Pichia pastoris*. Microb Cell Fact. 2009;8:29. 10.1186/1475-2859-8-29. 19490607 PMC2702363

[bib178_243_290825] McKinlay JB, Vieille C, Zeikus JG. Prospects for a bio-based succinate industry. Appl Microbiol Biotechnol. 2007;76:727–40. 17609945 10.1007/s00253-007-1057-y

[bib91] Melo NTM, Mulder KCL, Nicola AM et al. Effect of pyruvate decarboxylase knockout on product distribution using *Pichia pastoris* (*Komagataella phaffii*) engineered for lactic acid production. Bioeng. 2018;5:17. 10.3390/bioengineering5010017. PMC5874883 29462904

[bib92] Merico A, Ragni E, Galafassi S et al. Generation of an evolved *Saccharomyces cerevisiae* strain with a high freeze tolerance and an improved ability to grow on glycerol. J Ind Microbiol Biotechnol. 2011;38:1037–44. 10.1007/s10295-010-0878-3. 20878442

[bib93] Mironczuk AM, Dobrowolski A, Rakicka M et al. Newly isolated mutant of *Yarrowia lipolytica* MK1 as a proper host for efficient erythritol biosynthesis from glycerol. Process Biochem. 2015;50:61–8. 10.1016/j.procbio.2014.10.020.

[bib94] Mirończuk AM, Rzechonek DA, Biegalska A et al. A novel strain of *Yarrowia lipolytica* as a platform for value-added product synthesis from glycerol. Biotechnol Biofuels. 2016;9:180. 10.1186/s13068-016-0593-z. 27594914 PMC5009880

[bib95] Munch G, Sestric R, Sparling R et al. Lipid production in the under-characterized oleaginous yeasts, *Rhodosporidium babjevae* and *Rhodosporidium diobovatum*, from biodiesel-derived waste glycerol. Bioresour Technol. 2015;185:49–55. 10.1016/j.biortech.2015.02.051. 25747878

[bib96] Nikel PI, Ramirez MC, Pettinari MJ et al. Ethanol synthesis from glycerol by *Escherichia coli* redox mutants expressing adhE from Leuconostoc mesenteroides. J Appl Microbiol. 2010;109:492–504. 10.1111/j.1365-2672.2010.04668.x. 20149000

[bib97] Norbeck J, Blomberg A. Metabolic and regulatory changes associated with growth of *Saccharomyces cerevisiae* in 1.4 M NaCl. Evidence for osmotic induction of glycerol dissimilation via the dihydroxyacetone pathway. J Biol Chem. 1997;272:5544–54. 10.1074/jbc.272.9.5544. 9038161

[bib98] Ong KL, Fickers P, Lin CSK. Enhancing succinic acid productivity in the yeast *Yarrowia lipolytica* with improved glycerol uptake rate. Sci Total Environ. 2020;702:134911. 10.1016/j.scitotenv.2019.134911. 31733546

[bib99] Pagliaro M, Ciriminna R, Kimura H et al. From glycerol to value-added products. Angew Chem Int Ed Engl. 2007;46:4434–40. 10.1002/anie.200604694. 17471485

[bib100] Pahlman AK, Granath K, Ansell R et al. The yeast glycerol 3-phosphatases Gpp1p and Gpp2p are required for glycerol biosynthesis and differentially involved in the cellular responses to osmotic, anaerobic, and oxidative stress. J Biol Chem. 2001;276:3555–63. 10.1074/jbc.M007164200. 11058591

[bib101] Papanikolaou S, Aggelis G. Lipid production by *Yarrowia lipolytica* growing on industrial glycerol in a single-stage continuous culture. Bioresour Technol. 2002;82:43–9. 10.1016/S0960-8524(01)00149-3. 11848376

[bib102] Papanikolaou S, Beopoulos A, Koletti A et al. Importance of the methyl-citrate cycle on glycerol metabolism in the yeast *Yarrowia lipolytica*. J Biotechnol. 2013;168:303–14. 10.1016/j.jbiotec.2013.10.025. 24432372

[bib103] Papanikolaou S, Muniglia L, Chevalot I et al. *Yarrowia lipolytica* as a potential producer of citric acid from raw glycerol. J Appl Microbiol. 2002;92:737–44. 10.1046/j.1365-2672.2002.01577.x. 11966915

[bib104] Pavlik P, Simon M, Schuster T et al. The glycerol kinase (GUT1) gene of *Saccharomyces cerevisiae*: cloning and characterization. Curr Genet. 1993;24:21–5. 10.1007/BF00324660. 8358828

[bib105] Petelenz-Kurdziel E, Kuehn C, Nordlander B et al. Quantitative analysis of glycerol accumulation, glycolysis and growth under hyper osmotic stress. PLoS Comput Biol. 2013;9:e1003084. 10.1371/journal.pcbi.1003084. 23762021 PMC3677637

[bib106] Pettersson N, Filipsson C, Becit E et al. Aquaporins in yeasts and filamentous fungi. Biol Cell. 2005;97:487–500. 10.1042/BC20040144. 15966864

[bib107] Pezzella C, Giacobelli VG, Lettera V et al. A step forward in laccase exploitation: recombinant production and evaluation of techno-economic feasibility of the process. J Biotechnol. 2017;259:175–81. 10.1016/j.jbiotec.2017.07.022. 28751274

[bib108] Pinzi S, Garcia IL, Lopez-Gimenez FJ et al. The ideal vegetable oil-based biodiesel composition: a review of social, economical and technical implications. Energy Fuels. 2009;23:2325–41.

[bib109] Polburee P, Yongmanitchai W, Lertwattanasakul N et al. Characterization of oleaginous yeasts accumulating high levels of lipid when cultivated in glycerol and their potential for lipid production from biodiesel-derived crude glycerol. Fung Biol. 2015;119:1194–204. 10.1016/j.funbio.2015.09.002. 26615742

[bib110] Poli JS, da Silva MA, Siqueira EP et al. Microbial lipid produced by *Yarrowia lipolytica* QU21 using industrial waste: a potential feedstock for biodiesel production. Bioresour Technol. 2014;161:320–6. 10.1016/j.biortech.2014.03.083. 24727354

[bib111] Posas F, Chambers JR, Heyman JA et al. The transcriptional response of yeast to saline stress. J Biol Chem. 2000;275:17249–55. 10.1074/jbc.M910016199. 10748181

[bib112] Prabhu AA, Thomas DJ, Ledesma-Amaro R et al. Biovalorisation of crude glycerol and xylose into xylitol by oleaginous yeast *Yarrowia lipolytica*. Microb Cell Fact. 2020;19:121. 10.1186/s12934-020-01378-1. 32493445 PMC7271524

[bib113] Prista C, Loureiro-Dias MC, Montiel V et al. Mechanisms underlying the halotolerant way of *Debaryomyces hansenii*. FEMS Yeast Res. 2005;5:693–701. 10.1016/j.femsyr.2004.12.009. 15943004

[bib114] Raimondi S, Foca G, Ulrici A et al. Improved fed-batch processes with *Wickerhamomyces anomalus* WC 1501 for the production of D-arabitol from pure glycerol. Microb Cell Fact. 2022;21:179. 10.1186/s12934-022-01898-y. 36058916 PMC9442996

[bib115] Raimondi S, Rossi M, Leonardi A et al. Getting lipids from glycerol: new perspectives on biotechnological exploitation of *Candida freyschussii*. Microb Cell Fact. 2014;13:83. 10.1186/1475-2859-13-83. 24906383 PMC4064286

[bib116] Rakicka M, Lazar Z, Dulermo T et al. Lipid production by the oleaginous yeast *Yarrowia lipolytic*a using industrial by-products under different culture conditions. Biotechnol Biofuels. 2015;8:104. 10.1186/s13068-015-0286-z. 26213570 PMC4513389

[bib117] Rakicka-Pustułka M, Miedzianka J, Jama D et al. High value-added products derived from crude glycerol via microbial fermentation using *Yarrowia* clade yeast. Microb Cell Fact. 2021;20:195. 10.1186/s12934-021-01686-0. 34627248 PMC8502345

[bib118] Rakicka-Pustułka M, Mirończuk AM, Celińska E et al. Scale-up of the erythritol production technology—process simulation and techno-economic analysis. J Clean Prod. 2020;257:120533. 10.1016/j.jclepro.2020.120533.

[bib119] Ramirez-Castrillon M, Jaramillo-Garcia VP, Rosa PD et al. The oleaginous yeast *Meyerozyma guilliermondii* BI281A as a new potential biodiesel feedstock: selection and lipid production optimization. Front Microbiol. 2017;8:1776. 10.3389/fmicb.2017.01776. 29018411 PMC5614974

[bib120] Ranieri R, Candeliere F, Sola L et al. Production of arabitol from glycerol by immobilized cells of *Wickerhamomyces anomalus* WC 1501. Front Bioeng Biotechnol. 2024;12:1375937. 10.3389/fbioe.2024.1375937. 38659644 PMC11039890

[bib121] Remize F, Barnavon L, Dequin S. Glycerol export and glycerol-3-phosphate dehydrogenase, but not glycerol phosphatase, are rate limiting for glycerol production in *Saccharomyces cerevisiae*. Metab Eng. 2001;3:301–12. 10.1006/mben.2001.0197. 11676566

[bib122] Ren L, Liu Y, Xia Y et al. Improving glycerol utilization during high-temperature xylitol production with *Kluyveromyces marxianus* using a transient clustered regularly interspaced short palindromic repeats (CRISPR)/CRISPR-associated protein 9 system. Bioresour Technol. 2022;365:128179. 10.1016/j.biortech.2022.128179. 36283669

[bib123] Rendulić T, Perpelea A, Ortiz JPR et al. Mitochondrial membrane transporters as attractive targets for the fermentative production of succinic acid from glycerol in *Saccharomyces cerevisiae*. FEMS Yeast Res. 2024;24:foae009. 10.1093/femsyr/foae009. 38587863 PMC11014245

[bib124] Ronnow B, Kielland-Brandt MC. GUT2, a gene for mitochondrial glycerol 3-phosphate dehydrogenase of *Saccharomyces cerevisiae*. Yeast. 1993;9:1121–30. 10.1002/yea.320091013. 8256521

[bib125] Ruchala J, Kurylenko OO, Dmytruk KV et al. Construction of advanced producers of first- and second-generation ethanol in *Saccharomyces cerevisiae* and selected species of non-conventional yeasts (*Scheffersomyces stipitis, Ogataea polymorpha*). J Ind Microbiol Biotechnol. 2020;47:109–32. 10.1007/s10295-019-02242-x. 31637550 PMC6970964

[bib126] Rywińska A, Rymowicz W, Żarowska B et al. Comparison of citric acid production from glycerol and glucose by different strains of *Yarrowia lipolytica*. World J Microbiol Biotechnol. 2010;26:1217–24. 24026926 10.1007/s11274-009-0291-0

[bib127] Rywińska A, Tomaszewska-Hetman L, Lazar Z et al. Application of new *Yarrowia lipolytica* transformants in production of citrates and erythritol from glycerol. Int J Mol Sci. 2024;25:1475. 38338753 10.3390/ijms25031475 PMC10855631

[bib128] Sabir F, Loureiro-Dias MC, Prista C. Comparative analysis of sequences, polymorphisms and topology of yeasts aquaporins and aquaglyceroporins. FEMS Yeast Res. 2016;16:fow025. 10.1093/femsyr/fow025. 27001976

[bib129] Saenge C, Cheirsilp B, Suksaroge TT et al. Potential use of oleaginous red yeast *Rhodotorula glutinis* for the bioconversion of crude glycerol from biodiesel plant to lipids and carotenoids. Process Biochem. 2011;46:210–8. 10.1016/j.procbio.2010.08.009.

[bib130] Semkiv M, Kata I, Ternavska O et al. Overexpression of the genes of glycerol catabolism and glycerol facilitator improves glycerol conversion to ethanol in the methylotrophic thermotolerant yeast *Ogataea polymorpha*. Yeast. 2019;36:329–39. 10.1002/yea.3387. 30903803

[bib131] Semkiv MV, Ruchala J, Dmytruk KV et al. 100 years later, what is new in glycerol bioproduction?. Trends Biotechnol. 2020;38:907–16. 10.1016/j.tibtech.2020.02.001. 32584768

[bib132] Shen H, Gong Z, Yang X et al. Kinetics of continuous cultivation of the oleaginous yeast *Rhodosporidium toruloides*. J Biotechnol. 2013;168:85–9. 10.1016/j.jbiotec.2013.08.010. 23965273

[bib133] Shen T, Junyong Z, Xiushan Y. Evaluation of an adapted inhibitor-tolerant yeast strain for ethanol production from combined hydrolysate of softwood. Appl Energy. 2011;88:1792–6.

[bib134] Shi X, Park HM, Kim M et al. Isopropanol biosynthesis from crude glycerol using fatty acid precursors via engineered oleaginous yeast *Yarrowia lipolytica*. Microb Cell Fact. 2022;21:168. 10.1186/s12934-022-01890-6. 35986289 PMC9392242

[bib135] Signori L, Ami D, Posteri R et al. Assessing an effective feeding strategy to optimize crude glycerol utilization as sustainable carbon source for lipid accumulation in oleaginous yeasts. Microb Cell Fact. 2016;15:75. 10.1186/s12934-016-0467-x. 27149859 PMC4858929

[bib136] Souza KS, Ramos CL, Schwan RF et al. Lipid production by yeasts grown on crude glycerol from biodiesel industry. Prep Biochem Biotechnol. 2017;47:357–63. 27737603 10.1080/10826068.2016.1244689

[bib137] Souza KS, Schwan RF, Dias DR. Lipid and citric acid production by wild yeasts grown in glycerol. J Microbiol Biotechnol. 2014;24:497–506. 10.4014/jmb.1310.10084. 24473455

[bib138] Spier F, Buffon JG, Burkert CA. Bioconversion of raw glycerol generated from the synthesis of biodiesel by different oleaginous yeasts: lipid content and fatty acid profile of biomass. Indian J Microbiol. 2015;55:415–22. 10.1007/s12088-015-0533-9. 26543267 PMC4627960

[bib139] Sprague GF, Cronan JE. Isolation and characterization of *Saccharomyces cerevisiae* mutants defective in glycerol catabolism. J Bacteriol. 1977;129:1335–42. 10.1128/jb.129.3.1335-1342.1977. 191434 PMC235107

[bib140] Sriphuttha C, Boontawan P, Boonyanan P et al. Simultaneous lipid and carotenoid production via *Rhodotorula paludigena* CM33 using crude glycerol as the main substrate: pilot-scale experiments. Int J Mol Sci. 2023;24:17192. 10.3390/ijms242417192. 38139021 PMC10743220

[bib141] Stepanov N, Efremenko E. Immobilised cells of *Pachysolen tannophilus* yeast for ethanol production from crude glycerol. New Biotechnol. 2017;34:54–8. 10.1016/j.nbt.2016.05.002. 27184618

[bib142] Suwannarangsee S, Oh DB, Seo JW et al. Characterization of alcohol dehydrogenase 1 of the thermotolerant methylotrophic yeast *Hansenula polymorpha*. Appl Microbiol Biotechnol. 2010;88:497–507. 10.1007/s00253-010-2752-7. 20635082

[bib143] Swinnen S, Klein M, Carrillo M et al. Re-evaluation of glycerol utilization in *Saccharomyces cerevisiae*: characterization of an isolate that grows on glycerol without supporting supplements. Biotechnol Biofuels. 2013;6:157. 10.1186/1754-6834-6-157. 24209984 PMC3835864

[bib144] Szczepańczyk M, Rzechonek DA, Dobrowolski A et al. The overexpression of YALI0B07117g results in enhanced erythritol synthesis from glycerol by the yeast *Yarrowia lipolytica*. Molecules. 2021;26:7549. 10.3390/molecules26247549. 34946639 PMC8705655

[bib145] Takakuwa N, Nagahama S, Matsumura H et al. Efficient conversion of crude glycerol into triacylglycerol by the yeast *Pseudozyma* sp. TYC-2187 for biodiesel production. J Oleo Sci. 2013;62:605–12. 10.5650/jos.62.605. 23985490

[bib146] Tamas MJ, Luyten K, Sutherland FC et al. Fps1p controls the accumulation and release of the compatible solute glycerol in yeast osmoregulation. Mol Microbiol. 1999;31:1087–104. 10.1046/j.1365-2958.1999.01248.x. 10096077

[bib147] Tani Y, Yamada K. Glycerol metabolism in methylotrophic yeasts. Agric Biol Chem. 1987;51:1927–33.

[bib148] Tao Y, Bu C, Zou L et al. A comprehensive review on microbial production of 1,2-propanediol: micro-organisms, metabolic pathways, and metabolic engineering. Biotechnol Biofuels. 2021;14:216. 10.1186/s13068-021-02067-w. 34794503 PMC8600716

[bib149] ter Linde JJ, Liang H, Davis RW et al. Genome-wide transcriptional analysis of aerobic and anaerobic chemostat cultures of *Saccharomyces cerevisiae*. J Bacteriol. 1999;181:7409–13. 10.1128/JB.181.24.7409-7413.1999. 10601195 PMC94195

[bib150] Thiru M, Sankh S, Rangaswamy V. Process for biodiesel production from *Cryptococcus curvatus*. Bioresour Technol. 2011;102:10436–40. 10.1016/j.biortech.2011.08.102. 21930373

[bib151] Tsaruk A, Filip K, Sibirny A et al. Native and recombinant yeast producers of lactic acid: characteristics and perspectives. Int J Mol Sci. 2025;26:2007. 10.3390/ijms26052007. 40076630 PMC11900929

[bib152] Tsirigka A, Theodosiou E, Patsios SI et al. Novel evolved *Yarrowia lipolytica* strains for enhanced growth and lipid content under high concentrations of crude glycerol. Microb Cell Fact. 2023;22:62. 10.1186/s12934-023-02072-8. 37004109 PMC10067222

[bib153] van Zyl PJ, Prior BA, Gouws Kilian S. Regulation of glycerol metabolism in *Zygosaccharomyces rouxii* in response to osmotic stress. Appl Microbiol Biotechnol. 1991;36:369–74. 10.1007/BF00208158.

[bib154] Wang Y, Han Y, Liu C et al. Engineering *Yarrowia lipolytica* to produce L-malic acid from glycerol. ACS Synth Biol. 2024;13:3635–45. 10.1021/acssynbio.4c00445. 39444231

[bib155] Wang Z, Zhuge J, Cao Y et al. [The key enzymes of metabolisms of glycerol in *Candida glycerolgenesis*]. Wei Sheng Wu Xue Bao. 2000;40:180–7. 12548942

[bib156] Watanabe T, Watanabe I, Yamamoto M et al. A UV-induced mutant of *Pichia stipitis* with increased ethanol production from xylose and selection of a spontaneous mutant with increased ethanol tolerance. Bioresour Technol. 2011;102:1844–8. 10.1016/j.biortech.2010.09.087. 20947339

[bib157] Watanabe Y, Nagayama K, Tamai Y. Expression of glycerol 3-phosphate dehydrogenase gene (CvGPD1) in salt-tolerant yeast *Candida versatilis* is stimulated by high concentrations of NaCl. Yeast. 2008;25:107–16. 10.1002/yea.1550. 17914749

[bib158] Wei Z, Xu Y, Xu Q et al. Microbial biosynthesis of L-malic acid and related metabolic engineering strategies: advances and prospects. Front Bioeng Biotechnol. 2021;9:765685. 10.3389/fbioe.2021.765685. 34660563 PMC8511312

[bib159] Willke T, Vorlop K. Biotransformation of glycerol into 1,3-propanediol. Eur J Lipid Sci Technol. 2008;110:831–40. 10.1002/ejlt.200800057.

[bib160] Xiberras J, Klein M, de Hulster E et al. Engineering *Saccharomyces cerevisiae* for succinic acid production from glycerol and carbon dioxide. Front Bioeng Biotechnol. 2020;8:566. 10.3389/fbioe.2020.00566. 32671027 PMC7332542

[bib161] Xiberras J, Klein M, Nevoigt E. Glycerol as a substrate for *Saccharomyces cerevisiae* based bioprocesses—knowledge gaps regarding the central carbon catabolism of this ‘non-fermentable’ carbon source. Biotechnol Adv. 2019;37:107378. 10.1016/j.biotechadv.2019.03.017. 30930107

[bib162] Xu J, Zhao X, Wang W et al. Microbial conversion of biodiesel byproduct glycerol to triacylglycerols by oleaginous yeast *Rhodosporidium toruloides* and the individual effect of some impurities on lipid production. Biochem Eng J. 2012;65:30–6. 10.1016/j.bej.2012.04.003.

[bib163] Yang S, Pan X, Wang Q et al. Enhancing erythritol production from crude glycerol in a wild-type *Yarrowia lipolytica* by metabolic engineering. Front Microbiol. 2022;13:1054243. 10.3389/fmicb.2022.1054243. 36478868 PMC9720325

[bib164] Yang X, Jin G, Gong Z et al. Recycling biodiesel-derived glycerol by the oleaginous yeast *Rhodosporidium toruloides* Y4 through the two-stage lipid production process. Biochem Eng J. 2014;91:86–91. 10.1016/j.bej.2014.07.015.

[bib165] Yazdani SS, Gonzalez R. Anaerobic fermentation of glycerol: a path to economic viability for the biofuels industry. Curr Opin Biotechnol. 2007;18:213–9. 10.1016/j.copbio.2007.05.002. 17532205

[bib166] Yen HW, Yang YC, Yu YH. Using crude glycerol and thin stillage for the production of microbial lipids through the cultivation of *Rhodotorula glutinis*. J Biosci Bioeng. 2012;114:453–6. 10.1016/j.jbiosc.2012.04.022. 22627051

[bib167] Yen HW, Zhang Z. Effects of dissolved oxygen level on cell growth and total lipid accumulation in the cultivation of *Rhodotorula glutinis*. J Biosci Bioeng. 2011;112:71–4. 10.1016/j.jbiosc.2011.03.013. 21498112

[bib169] Yoshikawa J, Habe H, Morita T et al. Production of D-arabitol from raw glycerol by *Candida quercitrusa*. Appl Microbiol Biotechnol. 2014b;98:2947–53. 10.1007/s00253-013-5449-x. 24352735

[bib168] Yoshikawa J, Habe H, Morita T et al. Production of mannitol from raw glycerol by *Candida azyma*. J Biosci Bioeng. 2014a;117:725–9. 10.1016/j.jbiosc.2013.11.016. 24374122

[bib170] Yu KO, Jung J, Ramzi AB et al. Improvement of ethanol yield from glycerol via conversion of pyruvate to ethanol in metabolically engineered *Saccharomyces cerevisiae*. Appl Biochem Biotechnol. 2012;166:856–65. 10.1007/s12010-011-9475-9. 22161213

[bib171] Yu KO, Kim SW, Han SO. Engineering of glycerol utilization pathway for ethanol production by *Saccharomyces cerevisiae*. Bioresour Technol. 2010;101:4157–61. 10.1016/j.biortech.2010.01.066. 20149645

[bib172] Yu Z, Chang Z, Lu Y et al. Metabolic engineering of *Saccharomyces cerevisiae* for glycerol utilization. FEMS Yeast Res. 2023;23. 10.1093/femsyr/foad014. 36869777

[bib173] Yuzbasheva EY, Scarcia P, Yuzbashev TV et al. Engineering *Yarrowia lipolytica* for the selective and high-level production of isocitric acid through manipulation of mitochondrial dicarboxylate-tricarboxylate carriers. Metab Eng. 2021;65:156–66. 10.1016/j.ymben.2020.11.001. 33161142

[bib174] Zhang L, Nie MY, Liu F et al. Multiple gene integration to promote erythritol production on glycerol in *Yarrowia lipolytica*. Biotechnol Lett. 2021;43:1277–87. 10.1007/s10529-021-03113-1. 33797654

[bib175] Zhao L, Yu J, Zhang X et al. The ethanol tolerance of *Pachysolen tannophilus* in fermentation on xylose. Appl Biochem Biotechnol. 2010;160:378–85. 10.1007/s12010-008-8308-y. 18651246

[bib176] Zhou J, Yin X, Madzak C et al. Enhanced alpha-ketoglutarate production in *Yarrowia lipolytica* WSH-Z06 by alteration of the acetyl-CoA metabolism. J Biotechnol. 2012;161:257–64. 10.1016/j.jbiotec.2012.05.025. 22789476

[bib177] Ziuzia P, Janiec Z, Wróbel-Kwiatkowska M et al. Honey’s yeast—new source of valuable species for industrial applications. Int J Mol Sci. 2023;24:7889. 10.3390/ijms24097889. 37175595 PMC10178026

